# Dual‐Functional Silent‐Region SERS Nanoplatform for Real‐Time Bacterial Tracking and Wound Healing Therapy

**DOI:** 10.1002/advs.76574

**Published:** 2026-07-13

**Authors:** Hanbin Deng, Rui Wang, Dianqi Zhang, Wei Zhang, Jaebum Choo, Fabiao Yu, Shaowen Cheng

**Affiliations:** ^1^ NHC Key Laboratory of Tropical Disease Control, Engineering Research Center for Hainan Bio‐Smart Materials and Bio‐Medical Devices, Key Laboratory of Hainan Functional Materials and Molecular Imaging, School of Life Sciences and Medical Technology, Department of Wound Repair, The First Affiliated Hospital Hainan Medical University Haikou Hainan China; ^2^ Key Laboratory of Emergency and Trauma, Ministry of Education, Key Laboratory of Haikou Trauma, Key Laboratory of Hainan Trauma and Disaster Rescue Hainan Medical University Haikou China; ^3^ Department of Chemistry Chung‐Ang University Seoul South Korea

**Keywords:** photodynamic‐antibiotic combination therapy, real‐time monitoring, reducing recurrence risk, silent‐region sers, wound infection

## Abstract

Bacterial wound infections, particularly those caused by *Escherichia coli* and *Pseudomonas aeruginosa*, are difficult to treat due to biofilm formation, multidrug resistance, and chronic inflammation. Here, we report a multifunctional nanoplatform based on a surface‐enhanced Raman scattering (SERS) probe that enables simultaneous photodynamic therapy, antibiotic delivery, and real‐time infection monitoring. The probe comprises gold nanoparticles coated with Prussian blue, which acts both as a near‐infrared photosensitizer and a Raman reporter in the spectroscopically silent region, further loaded with colistin sulfate and stabilized with bovine serum albumin. Upon irradiation with a 650 nm laser, the probe produces reactive oxygen species, and, in combination with antibiotic release, achieves enhanced eradication of Gram‐negative bacteria (>90% reduction) using a significantly reduced antibiotic dose. The SERS signal in the silent region enables background‐free, in situ discrimination and quantification of bacterial load during treatment. In both normal and diabetic mouse wound models, the probe accelerates bacterial clearance, facilitates M2 macrophage polarization, and promotes angiogenesis and collagen deposition, thereby resulting in improved wound healing. Analysis of 36 clinical wound samples demonstrates sensitive and specific Gram‐positive and negative bacterial identification. This approach establishes a clinically translatable strategy for precise infection management, real‐time monitoring, and enhanced wound repair.

## Graphical abstract

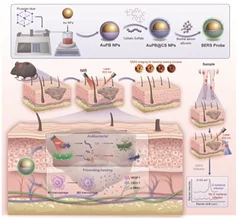

## 1 Introduction

Wound infections caused by pathogenic bacteria—particularly Gram‐negative strains such as *Escherichia coli* (*E. coli*) and *Pseudomonas aeruginosa* (*P. aeruginosa*)— pose a major clinical challenge due to biofilm formation, multidrug resistance (MDR), and persistent inflammation that delays healing [1]. Although conventional antibiotics are widely used, they often fail to prevent prolonged infection, tissue necrosis, and systemic complications, especially in the era of MDR pathogens [2, 3, 4, 5]. The limited pipeline of novel antibiotics further restricts therapeutic options, rendering monotherapy inadequate to meet clinical demands [6, 7]. In practice, recurrent wound infections can lead to progressive tissue destruction—such as osteomyelitis, tendon, or nerve damage—with permanent functional loss, elevated risk of systemic spread, increased treatment costs, and a profound impact on patients’ physical, psychological, and socioeconomic well‐being [2, 8, 9]. Effective strategies to prevent recurrence are therefore urgently needed.

Anatomically complex sites—such as deep cavities, sinus tracts, or regions adjacent to vital structures—are particularly prone to incomplete debridement due to limited visualization, difficulty in assessing tissue viability, and biofilm persistence [10, 11, 12]. Residual bacterial colonization is a major cause of reinfection, highlighting the need for thorough dead‐space management and effective local antimicrobial intervention. Photodynamic therapy (PDT), especially when combined with antibiotics, offers distinct advantages in this regard [13, 14, 15]. Under light activation, PDT rapidly disrupts bacterial membranes and damages intracellular components via reactive oxygen species (ROS) generation [16, 17]. When integrated with antibiotics, this dual mechanism can enhance antibacterial efficacy while reducing antibiotic dosage [18, 19]. Moreover, the spatially confined action of PDT minimizes off‐target effects and supports tissue repair.

Dynamic monitoring of bacterial load is critical for assessing infection control and wound healing. Standard methods such as colony‐forming unit (CFU) counting and polymerase chain reaction (PCR) face significant limitations: culture is slow (24–72 h) and detects mainly planktonic bacteria, whereas PCR—though sensitive—requires destructive sample preparation and cannot distinguish live from dead bacteria [20, 21]. In contrast, surface‐enhanced Raman scattering (SERS) enables rapid, non‐destructive, and in situ bacterial detection by exploiting plasmonic nanoscale enhancement [22, 23, 24, 25]. However, conventional SERS is often hindered by background interference from complex biological matrices. Silent‐region SERS overcomes this limitation by detecting Raman signals in the spectroscopically silent region (1800–2800 cm^−1^), effectively avoiding autofluorescence and endogenous molecular noise. This allows real‐time, interference‐free tracking of bacteria and therapeutic response.

To integrate both antibacterial and diagnostic functions, Prussian blue (PB) is an attractive multifunctional component. It serves as a near‐infrared photosensitizer for PDT and a Raman reporter in the silent region [27, 28, 29, 30]. PB efficiently generates singlet oxygen (^1^O_2_) under laser irradiation for ROS‐mediated bacterial killing, while its intrinsic Raman peak enables label‐free, background‐free SERS detection—streamlining probe construction and improving biocompatibility.

Here, we report a multifunctional silent‐region SERS probe that combines PDT, antibiotic therapy, and real‐time bacterial monitoring for treating complex and recurrent wound infections (Scheme 1). The probe consists of gold nanoparticles coated with PB, loaded with colistin sulfate (CS)—a potent anti‐Gram‐negative antibiotic—and stabilized with bovine serum albumin (BSA). Upon 650 nm laser irradiation, PB produces ROS for oxidative bacterial damage, while CS disrupts the bacterial outer membrane, achieving enhanced eradication. Simultaneously, PB's silent‐region Raman signal provides highly specific, non‐invasive in situ tracking of bacterial load and therapeutic progress. In both in vivo BALB/c and diabetic db/db mouse wound models infected with *E. coli*, *P. aeruginosa*, or mixed strains, this platform accelerates wound closure, eradicates bacterial burden, modulates macrophage polarization, and promotes collagen deposition, while demonstrating excellent biosafety. This integrated theranostic approach offers a promising strategy for precise infection control, real‐time monitoring, and recurrence prevention in challenging wound environments.

**SCHEME 1:**
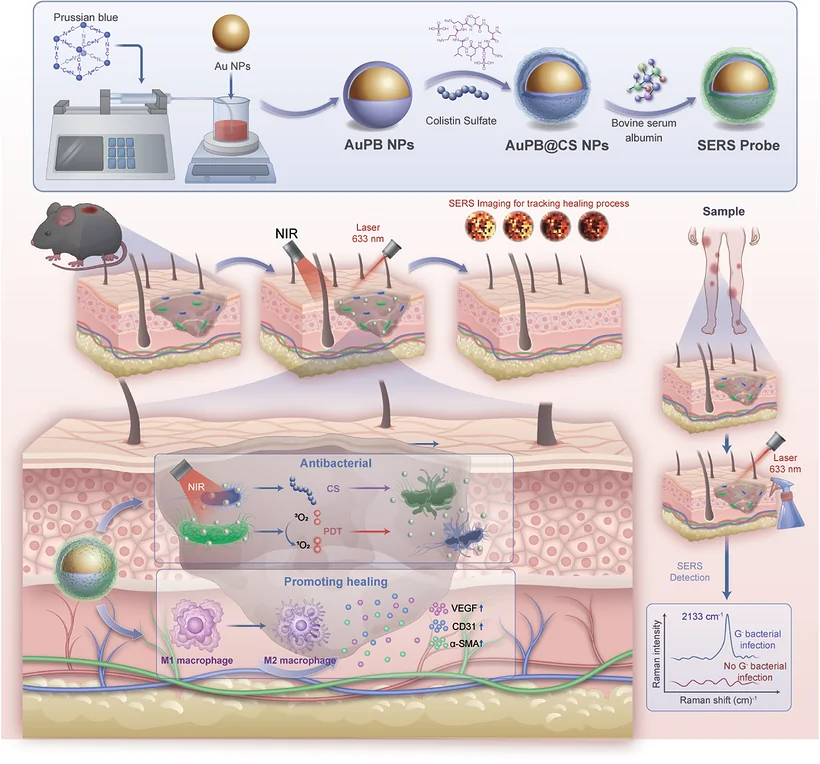
Schematic illustration of the preparation of silent‐region SERS probe and its combined treatments based on photodynamic therapy and antibiotics to promote healing of infected wounds and inhibit recurrence. Silent‐region SERS imaging, with a characteristic peak at 2133 cm^−1^, is used to track the healing process.

## 2 Results

### 2.1 Preparation and Characterization of SERS Probes

In this study, multifunctional silent‐region SERS probes were systematically prepared and comprehensively characterized to enable effective PDT‐antibiotic combined therapy and sensitive bacterial detection via SERS imaging. Briefly, gold nanoparticles (Au NPs) were synthesized using a modified seed‐growth method and were subsequently coated with PB to fabricate AuPB NPs [31]. These were then functionalized with a CS layer and stabilized with BSA to obtain the final SERS probes. As shown in Figure 1a, UV–vis absorption spectra revealed distinct changes after each modification step. Specifically, Au NPs displayed a characteristic plasmonic absorption peak at ∼ 520 nm, while AuPB NPs exhibited an additional broad absorption band at 700 nm, attributable to the PB coating [32]. In addition, the resulting SERS probe demonstrated further red shifts and changes in absorption intensity after CS and BSA modifications. Hydrodynamic size analysis showed a clear increase in particle size after each modification step, rising from 50 nm for Au NPs to about 75 nm for the SERS probe (Figure 1b). Furthermore, zeta potential measurements showed progressively more negative surface charges with each modification step, indicating improved colloidal stability and biocompatibility as shown in Figure 1c. To confirm the spectroscopic properties of the SERS probes, Raman spectroscopy was performed, which revealed a typical and strong Raman peak at 2143 cm^−1^ for the AuPB NPs and 2133 cm^−1^ for the SERS probe, whereas Au NPs exhibited only negligible signal in this region. This result demonstrates that PB served effectively as a silent‐region Raman reporter (Figure 1d).

**FIGURE 1:**
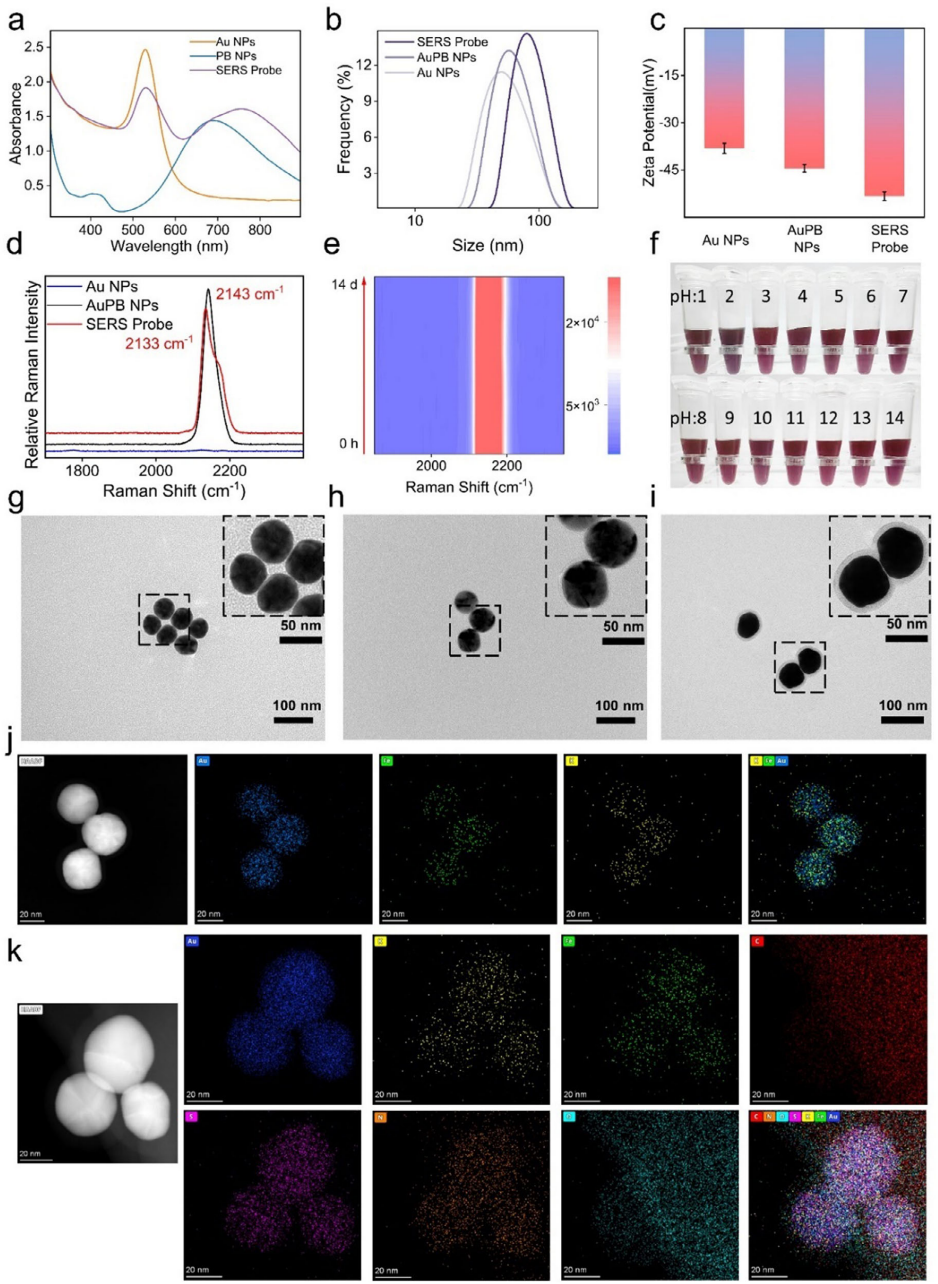
Preparation and characterization of SERS probes. (a) UV–vis absorption spectra of Au NPs, AuPB NPs and SERS probes. (b) Hydrodynamic size distributions and (c) zeta potentials of Au NPs, AuPB NPs and SERS probes. (d) SERS spectra of Au NPs, AuPB NPs and SERS probes. (e) Stability test of SERS signal intensity from SERS probes over 14 days. (f) Stability tests of SERS probes at different pH values ranging from 1 to 14. (g, h, i) TEM images of Au NPs, AuPB NPs and SERS probes. (j) EDS mapping of AuPB NPs (Au: blue, Fe: green, K: yellow). (k) EDS mapping of SERS probes (Au: blue, Fe: green, K: yellow, C: red, N: orange, O: cyan, S: purple).

The stability of Raman signal and its resistance to external environmental interference are critical parameters for physiological applications. Thus, the stability test of Raman signal intensity in fetal bovine serum demonstrated excellent signal retention over 14 days, indicating that the proposed probe shows minimal degradation and superior long‐term stability under physiological conditions (Figure 1e). In addition, as illustrated in Figure 1f, pH stability tests across a broad range from highly acidic (pH = 1) to strongly alkaline (pH = 14) demonstrated negligible aggregation of the SERS probe, further supporting its robustness and potential application in diverse biomedical environments. Additional results, including cytotoxicity and hemolysis assays of the SERS probes, confirmed their good biocompatibility (Figure S1). The morphology of SERS probe and its precursors were confirmed by transmission electron microscopy (TEM), which clearly showed that the spherical Au core nanoparticles were uniformly encapsulated by the PB shell and the additional organic coatings (Figure 1g–i). Energy‐dispersive spectroscopy (EDS) elemental mapping provided additional chemical verification of successful synthesis steps. Specifically, AuPB NPs displayed uniform distributions of gold (Au), iron (Fe), and potassium (K) (Figure 1j), confirming the consistent coating of the PB layer. The SERS probe showed additional elements—carbon (C), nitrogen (N), oxygen (O), and sulfur (S) signals— arising from organic biomolecules such as colistin sulfate and BSA (Figure 1k), affirming the integration of antibiotic and stabilizing agents. All these characteristic results confirm that the SERS probes are highly stable, robust, and suitable for sensitive, real‐time bacterial detection in complex biological environments.

### 2.2 Detection Performances of SERS Probes

We established bacterial suspensions with different bacteria concentrations (1×10^3^‐10^7^ CFU/mL) and incubated with the SERS probes under the optimized conditions. The SERS signal intensity at 2133 cm^−1^ was recorded and compared with the corresponding CFU counts. The probe exhibited a wide response range from 1×10^3^ to 1×10^7^ CFU/mL, with the limits of detection of 127 CFU/mL and 143 CFU/mL for E. coli and P. aeruginosa, respectively (Figure 2a–d). The results exhibited that a positive correlation between SERS intensity and viable bacterial number within the tested concentration range, which indicated that the SERS signals could be utilized to quantitatively detect Gram‐negative bacteria burden.

**FIGURE 2:**
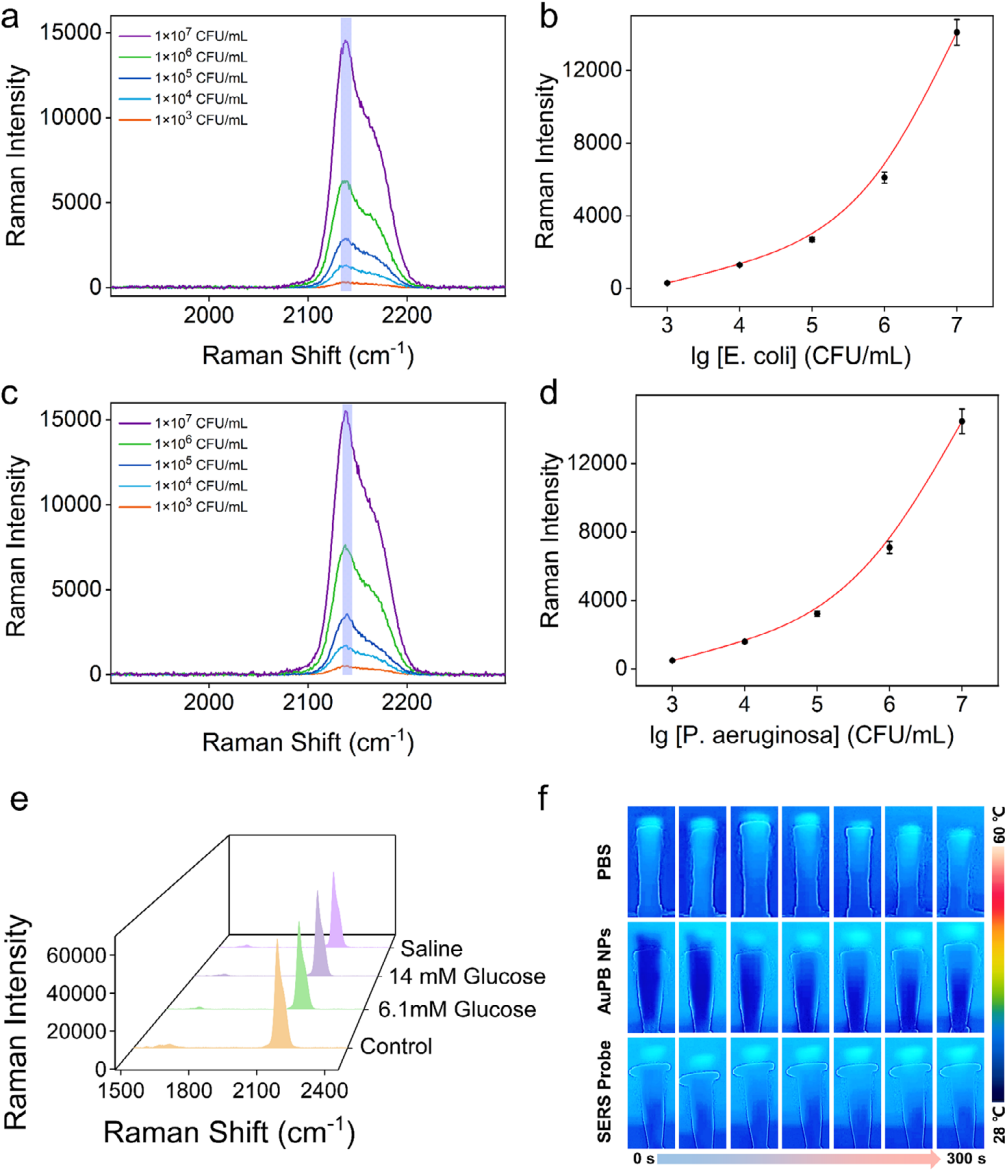
In vitro bacterial detection performance of SERS probe. (a) and (c) In vitro SERS spectra obtained after incubating different concentrations of E. coli and P. aeruginosa with SERS probe with 633 nm laser irradiation. (b) and (d) the calibration curves of the Raman peak intensity at 2133 cm^−1^ and the different concentrations of E. coli and P. aeruginosa, respectively. (e) The stability of the SERS probe under different physiological conditions was tested, including detecting changes in the SERS signal of the SERS probe in PBS, physiological saline, 6.1 mM and 14 mM glucose. (f) Temperature changes of SERS probe over different time periods under laser irradiation. Infrared thermal images of SERS probe, AuPB NPs, and PBS solution taken over 5 min under 650 nm near‐infrared laser irradiation.

Considering that diabetic wounds are characterized by the high glucose microenvironment, we further evaluated the effect of glucose on the performance of the SERS probe (Figure 2e). The SERS probe was incubated under normal‐glucose (6.1 mM) high‐glucose (14 mM) conditions, and the typical Raman signal at 2133 cm^−1^ was monitored. No obvious changes could be observed under high‐glucose conditions, which indicated that glucose did not significantly interfere with the silent‐region SERS signals of the region. Since PB has potential photothermal conversion ability, we monitored the temperature changes of the SERS probe solution under 650 nm laser irradiation at 200 mW/cm^2^ for 5 min (Figure 2f). The results showed no significant temperature elevation under the current conditions, which indicated that there was no obvious photothermal therapy effect.

### 2.3 Assessment of the PDT Properties of SERS Probes and Incubation Optimization

The time‐resolved fluorescence spectra demonstrated the progressive generation of singlet oxygen (^1^O_2_) by the SERS probes under laser irradiation, as evidenced by the increasing emission intensity at 528 nm over a 20‐min period (Figure 3a). The linear relationship between fluorescence intensity and time confirmed the sustained production of ^1^O_2_, a key reactive species for antimicrobial activity. To calculate the encapsulation efficiency of colistin sulfate (CS) on AuPB NPs, a calibration curve was constructed for absorbance values at 562 nm vs. CS concentration in the range of 0∼200 µg/mL (Figure 3c). The encapsulation efficiency was calculated to be 66.1% based on the amount of CS in the supernatant and the encapsulated content.

**FIGURE 3:**
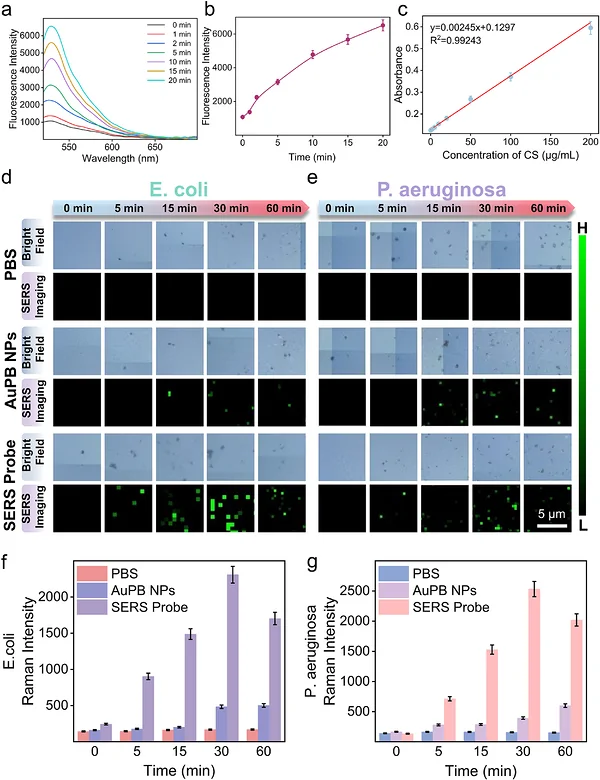
Performance of SERS probes. (a) Fluorescence spectra of SERS probes treated with the SOSG probe over 20 min (λ_ex_: 488 nm, λ_em_: 520–700 nm). (b) Changes in fluorescence intensity at 528 nm over 20 min for the SERS probes with the SOSG probes. (c) Calibration curve of colistin sulphate (CS) based on the UV–vis absorption band at 562 nm. (d, e) PBS, AuPB NPs and SERS probes were co‐incubated with *E. coli* (d) and *P. aeruginosa* (e) for 0, 5, 15, 30 and 60 min, and representative bright‐field and SERS images at 2133 cm^−1^ were obtained (scale bar: 10 µm). (f, g) Quantification of SERS signal intensity at different time points for PBS, AuPB NPs and SERS probes after incubation with *E. coli* (f) and *P. aeruginosa* (g), respectively. (scale bar: 5 µm).

To further evaluate the ^1^O_2_ production by the SERS probes under 650 nm laser irradiation, the commercial ^1^O_2_ fluorescent probe Singlet Oxygen Sensor Green (SOSG) was employed, which is insensitive to hydroxyl radicals and superoxide [33]. In the presence of ^1^O_2_, green fluorescence was emerged, with the intensity at 528 nm rapidly increasing within 20 min, demonstrating continuous ^1^O_2_ production from the SERS probes (Figure 3a,b). CS belongs to the class of polypeptide antibiotics, which are natural narrow‐spectrum antibiotics obtained from the culture medium of *Bacillus polymyxa*. Its polycation ring interacts with phosphate groups on the outer membrane of Gram‐negative bacteria, resulting in increased permeability, leakage of intracellular molecules such as purines and pyrimidines, and ultimately bacterial swelling, lysis, and death [34]. CS exhibits strong antibacterial activity against most Gram‐negative strains but shows minimal activity against Gram‐positive bacteria.

To optimize the incubation time between SERS probes and bacteria for maximal antibacterial efficacy, SERS imaging was conducted at different time points (0, 5, 15, 30 and 60 min) to track signal changes from *E. coli* and *P. aeruginosa* during incubation. The SERS probes exhibited their highest signal intensity at 2133 cm^−1^ after 30 min, whereas the SERS signals gradually decreased by 60 min. This reduction likely arises from the antibacterial activity of CS immobilized on the probe surface. Therefore, 30 min was determined to be the optimal incubation time for treating the bacteria with the SERS probes. The stability and selectivity of the SERS peak at 2133 cm^−1^ highlight the probes’ dual functionality as antimicrobial agents and real‐time biosensors (Figure 3f,g). Moreover, the sustained SERS signal intensity during bacterial inactivation suggests that the probes retain their structural integrity under operational conditions, a critical feature for long‐term monitoring.

### 2.4 In Vitro Antibacterial Efficacy

With the excellent photodynamic and antimicrobial properties of the SERS probes, their antibacterial activity in bacterial culture in vitro was further confirmed under 650 nm laser irradiation. *E. coli* and *P. aeruginosa* were selected as model strains representing typical Gram‐negative bacteria. The concentration‐dependent antibacterial activity of SERS probes was first tested (Figure 4a–d). The minimum inhibitory concentration (MIC) was determined using a modified broth microdilution assay. Various concentrations of the SERS probe (0, 0.094, 0.187, 0.375, 0.750 and 1.500 nM) were mixed with the bacterial solution in 96‐well plates and irradiated with a 650 nm laser (200 mW/cm^2^, 5 min). Then, the mixture of SERS probes and bacterial solution was transferred to the Luria‐Bertani (LB) broth and incubated for 24 h. medium was used as the blank control, and the bacterial solution without any treatment served as the negative control. The minimum concentration without visible turbidity was regarded as the MIC at the selected bacterial concentration. As shown in Figure 4a–d, when the concentrations of SERS probes, were as low as 0.375 nM for *E. coli* and 0.187 nM for *P. aeruginosa*, no obvious turbidity was observed. Thus, the MIC were determined to be 0.375 nM and 0.187 nM, respectively, which were also used to evaluate the antibacterial performance.

**FIGURE 4:**
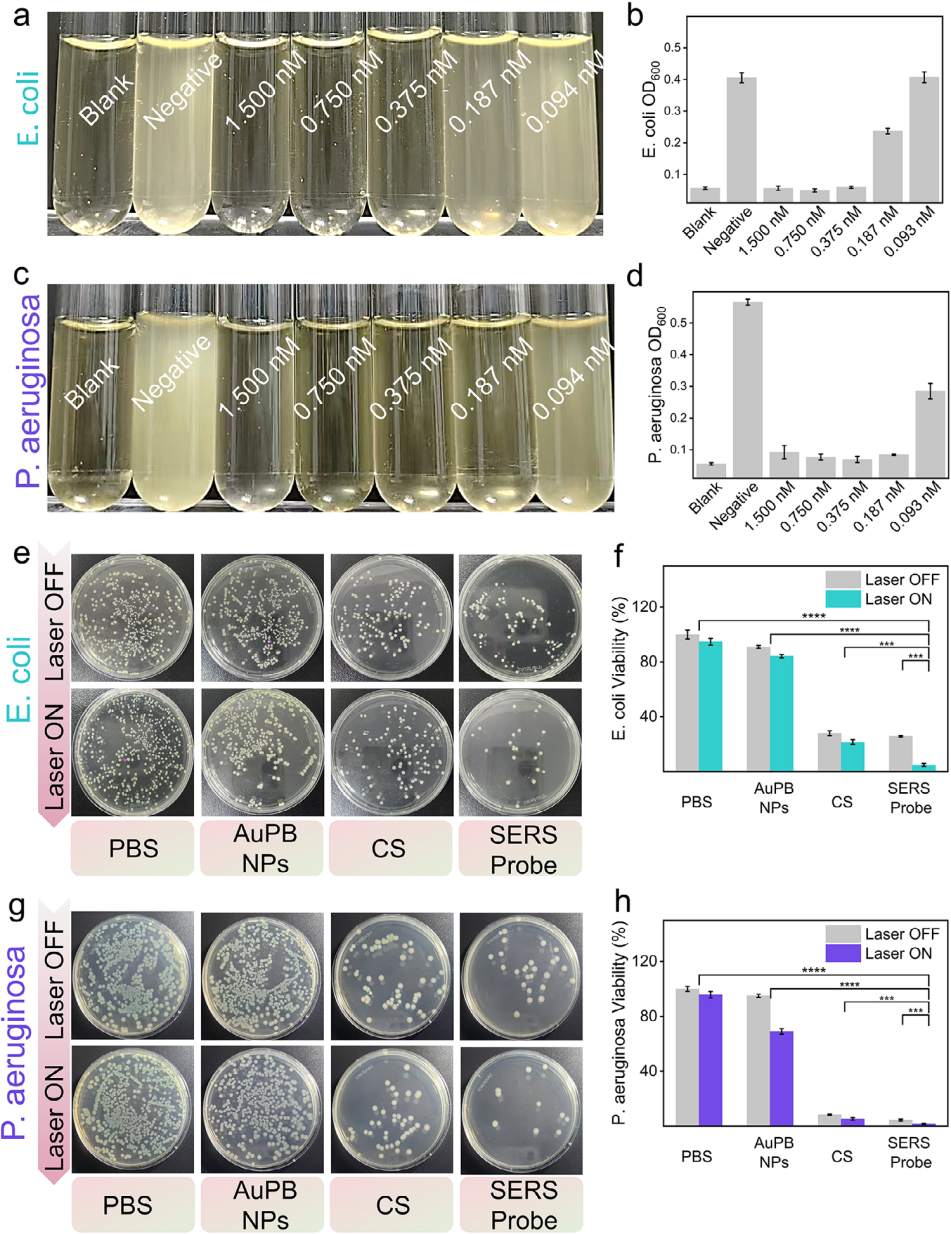
In vitro antibacterial efficacy of SERS probes. Photos of (a) *E. coli* and (b) *P. aeruginosa* cultured overnight in LB broth medium after different treatments: blank, negative control, and SERS probes (1.5, 0.75, 0.375, 0.187 and 0.094 nM) under laser irradiation for 5 min (650 nm, 200 mW/cm^2^). (b, d) OD_600_ values of the corresponding cultures shown in (a) and (c), respectively. Representative colony formation images of (e) *E. coli* and (g) *P. aeruginosa* treated with PBS, AuPB NPs, CS and SERS probes with the corresponding bacterial viability is measured in (f) and (h), respectively.

In addition, to further confirm the combined effect of photodynamic therapy and the colistin‐loaded SERS probes, colony formation assays were performed under different treatments. As shown in Figure 4e–g, the SERS probes achieved over 99% reduction in bacteria viability for both strains, significantly outperforming AuPB NPs (20%–30% reduction). These results indicate that the probe's antibacterial action arises from a combined mechanism: laser‐triggered ^1^O_2_ generation induces oxidative membrane damage, while CS enhances bacterial susceptibility. The absence of antibacterial activity in non‐irradiated controls underscored the light‐dependent nature of the system. Moreover, the dose‐response further supported a ROS‐mediated antibacterial mechanism, as higher probe concentrations enhanced ^1^O_2_ production, leading to accelerated bacterial inactivation.

To further evaluate the therapeutic potential of the SERS probe against biofilm‐associated infection, mature P. aeruginosa films were established in vitro and treated with PBS, AuPB NPs, CS, and SERS probes, with or without 650 nm laser irradiation (Figure S2a–f). Crystal violet staining showed that the SERS probes with laser irradiation significantly reduced biofilm biomass. In addition, fluorescence imaging further revealed the reduced biofilm thickness, while the optical density at 590 nm demonstrated that the extensive bacterial death after treatment (Figure S2g,h). These results indicated that the SERS probe could damage biofilm‐associated bacteria and partially disrupt the biofilm matrix under laser irradiation.

To further evaluate the antibiotic delivery behavior of the SERS probe, CS release kinetics were investigated in wound‐like media containing proteins and physiologically relevant ionic strength, with 650 nm laser irradiation (Figure S3a). In addition, the CS dose used in the free antibiotic control was matched to the amount of CS loaded in the SERS probe. The released CS was quantified at predetermined time points (0, 3, 6, 12, 24, 48, and 72 h). The SERS probe showed sustained CS release under wound‐mimicking conditions, which demonstrated that CS was not merely adsorbed as an immediately detachable surface coating (Figure S3b). To confirm the bioactivity of released CS, release media collected from SERS probes were applied to Gram‐negative bacteria and evaluated by CFU counting analysis (Figure S3c–e). The released CS retained antibacterial activity against E. coli and P. aeruginosa, which further confirmed the functional antibiotic delivery capability of the SERS probe.

### 2.5 Investigation of the Antibacterial Mechanism

Furthermore, we investigated the photodynamically induced antibacterial activity of the SERS probe against *E. coli* and *P. aeruginosa* by monitoring ROS generation and bacterial viability under laser irradiation. As shown in Figure 5a, live/dead bacterial staining revealed a distinct bactericidal effect following treatment with the SERS probe under 650 nm irradiation (200 mW/cm^2^, 5 min). Green fluorescence (SYTO 9) represents live bacteria, whereas red fluorescence (propidium iodide, PI) indicates dead bacteria. In the absence of laser irradiation, all control groups, including PBS, AuPB NPs, CS, and SERS probes, exhibited negligible cytotoxicity, reflected by predominantly green fluorescence signals. Upon irradiation, however, the SERS probe group displayed intense red fluorescence, confirming extensive membrane disruption and cell death in both bacterial strains. Notably, *P. aeruginosa* exhibited slightly higher resistance than *E. coli*, suggesting that the photodynamic action of the SERS probe plays a major role in ROS‐mediated bacterial inactivation.

**FIGURE 5:**
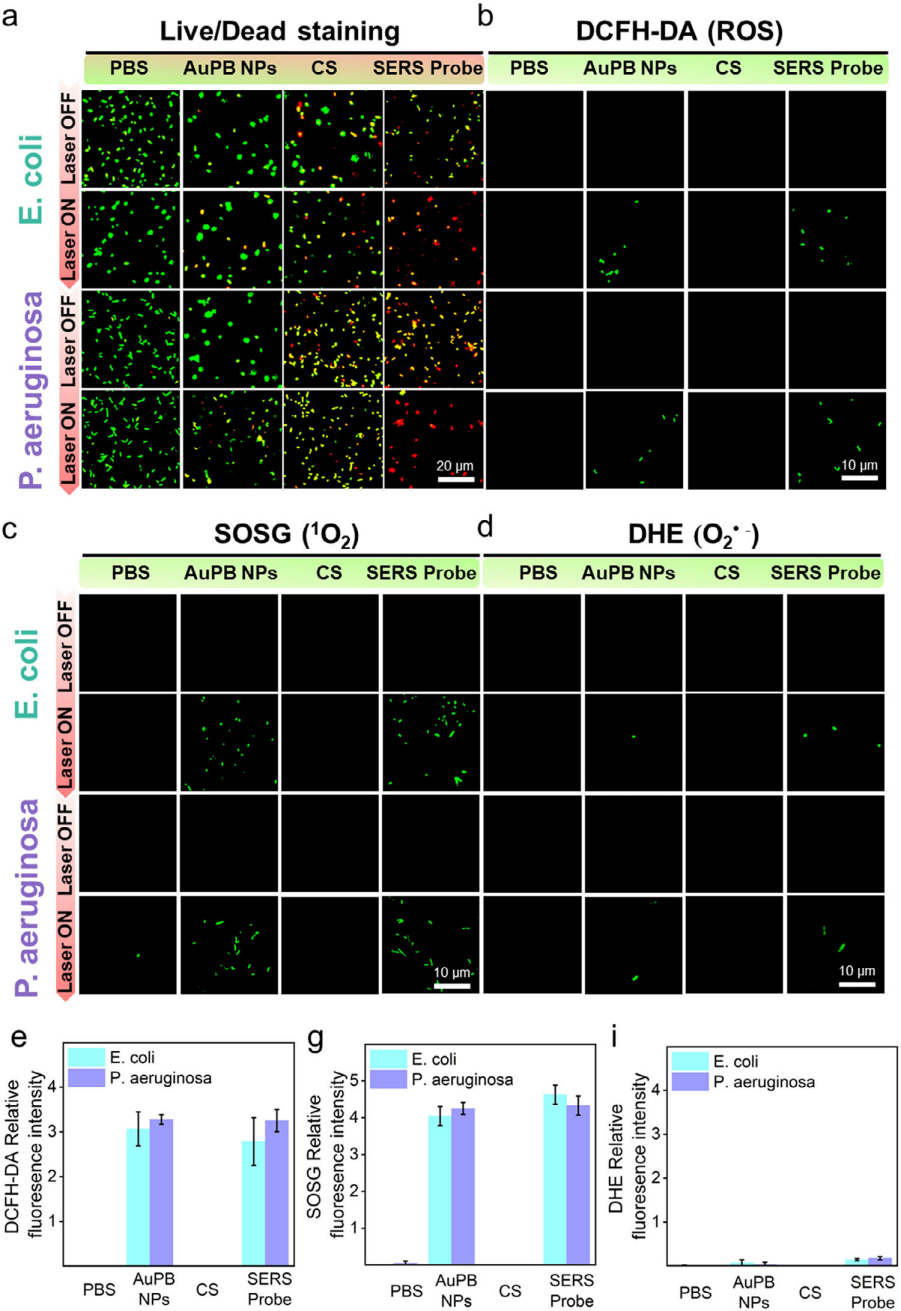
Fluorescent imaging of ROS generated in bacteria. (a) Representative fluorescence images of live/dead bacterial staining following treatment with PBS, AuPB NPs, CS and SERS probes, with or without laser irradiation (650 nm, 200 mW/cm^2^, 5 min) for *E. coli* and *P. aeruginosa* (green: SYTO 9, λ_ex_: 488 nm, λ_em_: 510 nm–550 nm; red: PI, λ_ex_: 561 nm, λ_em_: 617 nm–667 nm). Scale bar: 20 µm. (b–d) DCFH‐DA, SOSG, DHE probes were used to indicate the production of ROS, ^1^O_2_, O_2_^.−^, respectively, after treatment with PBS, AuPB NPs, CS and SERS probes, with or without laser irradiation. Scale bar: 10 µm. (e–g) The relative fluorescence intensities from (b–d) were quantified.

To further elucidate the mechanism of photodynamic antibacterial effect, specific fluorescent probes were employed for different types of ROS. DCFH‐DA was used to detect general ROS, SOSG for singlet oxygen (^1^O_2_), and DHE for superoxide anion (O_2_ **^·−^**). As shown in Figure 5b–d, distinct green fluorescence signals were observed only in the SERS probe under laser irradiation, with SOSG yielding the strongest response, demonstrating that singlet oxygen is the predominant ROS species. Quantitative fluorescence analysis confirmed the significant elevation of ^1^O_2_ under SERS probe irradiation relative to PBS, AuPB NPs, and CS (Figure 5e–g). Although DCFH‐DA and DHE also showed increased signals, the comparatively greater enhancement in SOSG confirmed that ^1^O_2_ plays a dominant role in this system. Importantly, all groups without irradiation exhibited minimal ROS generation, underscoring the strict light‐dependent controllability of this system. Collectively, these results demonstrate that the SERS probe acts as an effective antibacterial platform through ROS‐mediated killing, thereby offering a non‐antibiotic strategy to combat drug‐resistant pathogens.

To further explore the antibacterial mechanism, three complementary analyses were performed. First, the optical density at 260 nm (OD_260_), reflecting nucleic acid leakage, was measured for *E. coli* and *P. aeruginosa* after treatment with PBS, AuPB NPs, CS, and SERS probes, with or without laser irradiation (650 nm, 200 mW/cm^2^, 5 min). As shown in Figure 6a,b, the SERS probe combined with irradiation resulted in the maximum increase in OD_260_, indicating enhanced nucleic acid leakage and severe membrane damage. In both bacterial strains, treatments with AuPB NPs or CS caused moderate leakage, while PBS had minimal effect.

**FIGURE 6:**
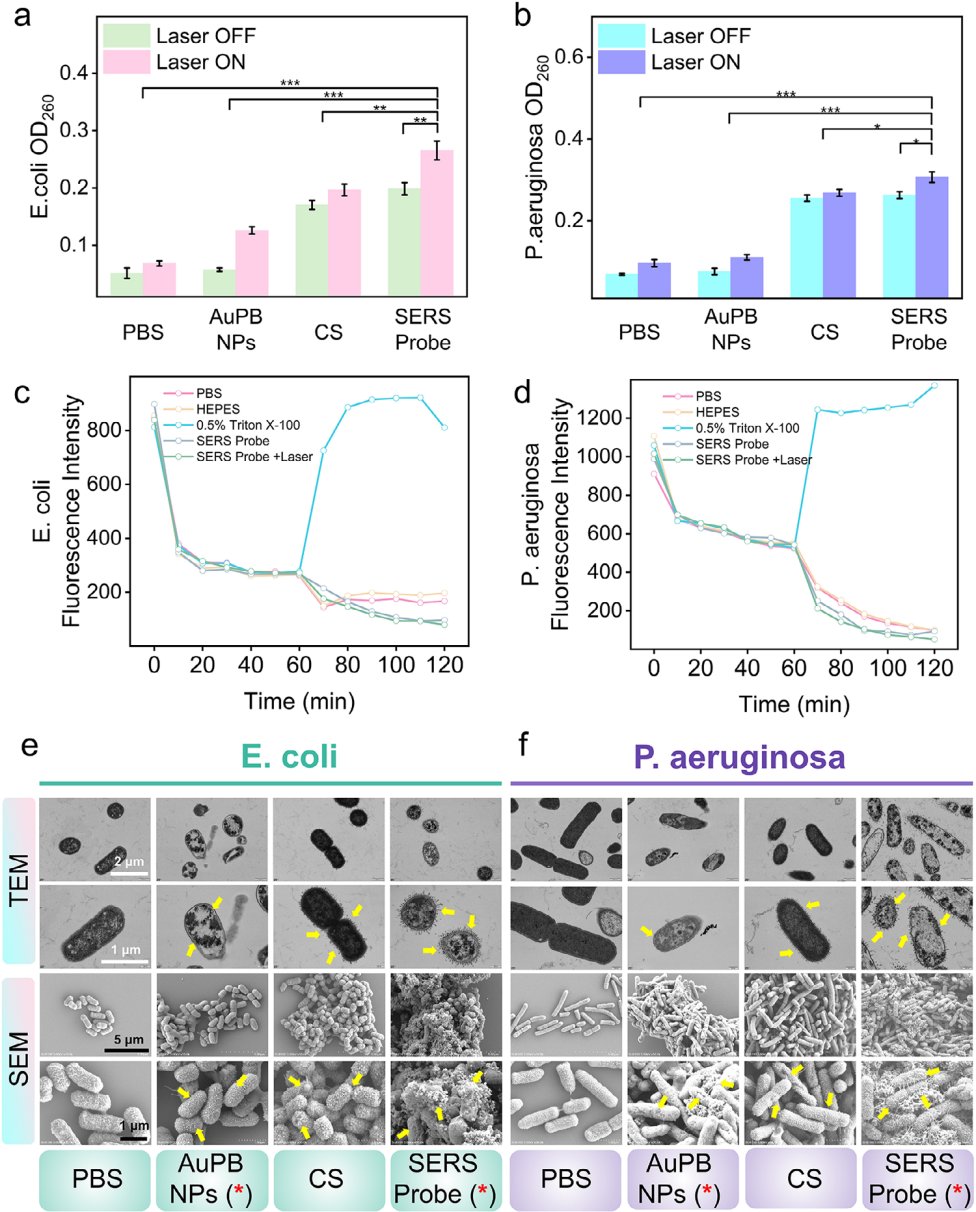
Investigation of the antibacterial mechanism. OD_260_ values were used to evaluate internal nucleic acid leakage in (a) *E. coli* and (b) *P. aeruginosa* after treatment with PBS, AuPB NPs, CS and SERS probes, with or without laser irradiation (650 nm, 200 mW/cm^2^, 5 min). Changes in the fluorescence intensity of the DiSC3(5) probe are shown for (c*) E. coli* and (d) *P. aeruginosa* following treatment with PBS, HEPES, 0.5% Triton, and SERS probes under laser irradiation (650 nm, 200 mW/cm^2^, 5 min). (e, f) TEM and SEM images of *E. coli* and *P. aeruginosa* after treatment with PBS, AuPB NPs, CS and SERS probes. *indicates laser irradiation (650 nm, 200 mW/cm^2^, 5 min).

Second, the changes in bacterial membrane potential were evaluated using the DiSC3(5) fluorescent probe. As depicted in Figure 6c,d, the addition of 0.5% Triton X‐100 led to a marked increase in fluorescence intensity. Conversely, PBS, HEPES, and SERS probes reduced fluorescence signals, which further decreased under irradiation in the SERS probe group. This decrease corresponds to membrane hyperpolarization rather than depolarization [35]. Hyperpolarization of the bacterial membrane may result from potassium efflux or tyrosine kinase‐A (TrkA) inactivation. This altered membrane potential disrupts ionic balance, leading to eventual membrane damage [36].

Third, ultrastructural analyses using TEM and SEM provided direct visualization of bacterial damage. As shown in Figure 6e,f, control bacteria (PBS group) exhibited smooth intact membranes, while AuPB NPs and CS treatments caused only minor membrane deformation. In contrast, bacteria treated with the SERS probe and exposed to laser irradiation exhibited significant structural damage, including ruptured membranes, cytoplasmic leakage, and cellular collapse. TEM revealed dense aggregates and vacuolated cytoplasm in the laser irradiated SERS probe group, consistent with intracellular content leakage and ROS‐mediated damage. SEM showed visibly lysed cells with surface irregularities and pore formation as highlighted by yellow arrows. All these results demonstrated that the proposed SERS probe induced the combined photodynamic and antibacterial effect characterized by ROS generation, membrane hyperpolarization, and structural disaggregation.

### 2.6 In Vivo Antibacterial Efficacy

To access the antibacterial application of the SERS probe in vivo, wound infection models were established in BALB/c mice using *E. coli* and *P. aeruginosa* as representative Gram‐negative pathogens. A standardized 8‐mm excisional wound was created on the dorsal skin of female BALB/c mice (18–22 g). Wound areas were recorded every other day and analyzed using Image J software. The infected mice were randomly divided into five treatment groups: control (no treatment), PS, AuPB NPs, CS, and SERS probe groups with laser irradiation. As shown in Figure 7a,b, control and PBS groups exhibited delayed epithelial closure and persistent inflammation through day 8, while the SERS probe treatment resulted in rapid reduction of wound size, alleviation of inflammation, and near‐complete closure by day 8. Stimulated wound healing rate diagrams corroborated this accelerated recovery.

**FIGURE 7:**
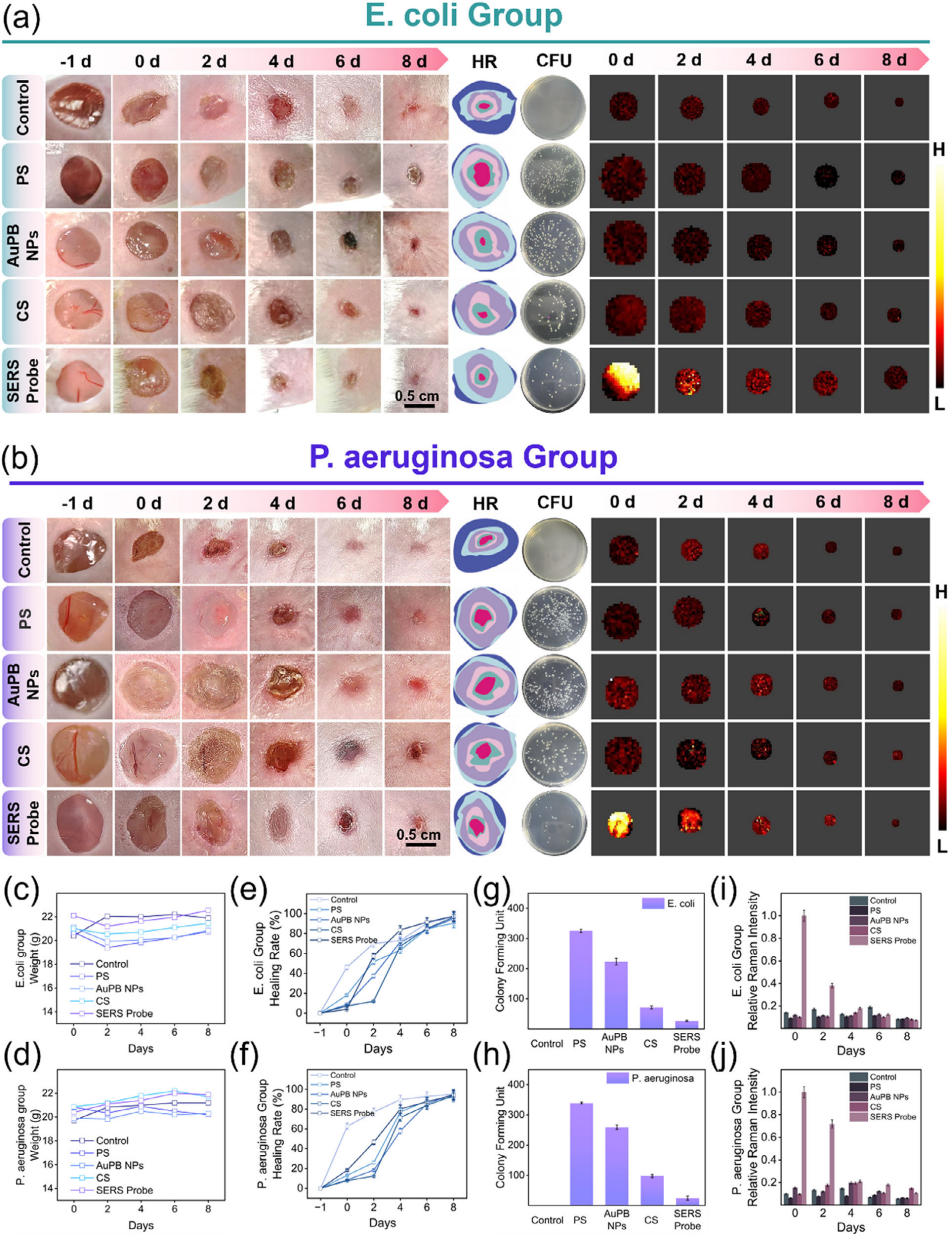
In vivo antibacterial efficacy of the SERS probe. The wounds of BALB/c mice were inoculated with (a) *E. coli* and (b) *P. aeruginosa* and divided into five groups: control, PS, AuPB NPs, CS and SERS probes with laser irradiation. Representative wound images after different treatments, simulated images of healing rate (HR), LB agar plate images, and SERS mapping images of bacterial colonies following different treatments are shown. (c, d) Body weight, (e, f) healing rate, (g, h) plate colony‐forming unit (CFU) count, and (i, j) quantitative SERS imaging results of the BALB/c mouse wound infection model with *E. coli* and *P. aeruginosa*, respectively. Scar bar: 0.5 cm.

Colony formation assays revealed a substantial reduction in bacterial loads in the SERS probe group, which was consistent with the wound closure data. SERS imaging further verified progressive bacterial clearance over time, showing strong SERS signals at baseline (day 0) and almost complete disappearance by day 8 in the SERS probe group. In contrast, residual Raman signals persistent in other treatment groups, particularly PS and AuPB NPs, highlighting their insufficient antibacterial efficacy.

To ensure the biosafety and systemic tolerance of the treatments, changes in body weights were monitored in all groups (Figure 7c,d). All groups maintained relatively stable weights without significant differences, demonstrating that neither the SERS probe nor the other treatments induced systemic toxicity. Wound healing rates were quantified, revealing that the SERS probe group exhibited the most rapid and consistent wound closure (Figure 7e,f). In both *E. coli* and *P. aeruginosa* infection models, the SERS probe outperformed all other treatments. These results demonstrated not only efficient bacterial activity but also accelerated tissue regeneration, possibly facilitated by the reduction of local inflammation due to efficient bacterial clearance. The AuPB NP group showed moderate wound‐healing improvement, confirming the critical role of the PB molecules in PDT. Furthermore, the quantitative analysis of colony formation and SERS signal intensity validated the therapeutic efficacy of the SERS probe (Figure 7g–j). Colony counts were markedly reduced in the SERS probe group for both bacterial strains, reflecting the highest antibacterial efficiency compared with other treatments (Figure 7g,h). Consistently, SERS signal intensity declined sharply over time in the SERS probe group, reflecting the clearance of bacteria from the wound sites. Collectively, these findings indicate that the SERS probe not only enables non‐invasive, real‐time monitoring of bacterial infection but also exerts a potent antibacterial effect under laser irradiation.

Infected wounds are a common and serious complication of diabetes. Hyperglycemia damages nerves and vessels, impairs immune function, and delays wound healing, making wounds susceptible to secondary infections. These infections can easily spread to deeper tissues, causing osteomyelitis or sepsis, which significantly increases the risk of amputation or death. To assess the therapeutic performance of the SERS probe in this context, full thickness wounds were generated in db/db mice and infected with *E. coli* or *P. aeruginosa*. Mice were then treated with PS, AuPB NPs, CS, or SERS probes with laser irradiation, with untreated mice serving as controls. As shown in Figure S4a,b, wounds in the control and PS groups exhibited pronounced inflammation, eschar formation, and delayed repair over 8 days. By contrast, the SERS probe group displayed rapid wound contraction and improved tissue recovery. Stimulated healing maps further visualized this trend, showing nearly closed wounds by day 8 in the SERS probe group, while other groups showed incomplete repair. Colony assays confirmed substantial bacterial clearance only in the SERS probe group, consistent with efficiency shown by SERS imaging, which revealed a progressive reduction in SERS signal intensity. Body weight measurements also confirmed systemic safety, as all groups maintained stable weights (Figure S4c,d). Healing rate quantification supported these findings, with the SERS probe achieving the highest wound closure rates in both bacterial infection models (Figure S4e,f). The CS group showed modest benefit due to antibiotic action, whereas AuPB NPs had limited effect, indicating that the plasmonic core alone was insufficient for complete bacterial elimination. Notably, diabetic db/db mice, characterized by defective healing and infection vulnerability, provided a clinically relevant and stringent evaluation of SERS probe efficacy [37]. Moreover, bacterial clearance was validated by colony counts and SERS signal quantification (Figure S4g–j), which demonstrated significantly lower bacterial survival and steadily declining Raman signals only in the SERS probe group. These results emphasized the value of SERS imaging as a non‐invasive, real‐time tool to monitor therapeutic effectiveness.

In polymicrobial infections, distinct bacterial species often form combined biofilms that enhance virulence and antimicrobial resistance via metabolic cooperation (aerobic/anaerobic symbiosis) and gene exchange. Such infections accelerate tissue necrosis, chronic suppuration, and increase the risk of systemic spread. To evaluate the theragnostic potential of the SERS probe in this setting, full‐thickness wounds were simultaneously infected with *E. coli* and *P. aeruginosa* in BALB/c and db/db mice. Animals were grouped into untreated control, PS, AuPB NPs, CS, and SERS probes with 650 nm laser irradiation. As shown in Figure S5a,b, the control and PS groups exhibited persistent inflammation, necrosis, and impaired wound closure. In contrast, the SERS probe group demonstrated rapid contraction and visible healing improvement by day 8. Healing‐rate diagrams further confirmed superior recovery in the SERS probe group in both mouse models. LB agar plate assays revealed substantial bacterial residuals in the control, PS, and AuPB NP groups, whereas CS led to partial reduction. Notably, the SERS probe induced near‐complete bacterial clearance. SERS imaging validated these outcomes, showing initially high Raman signals (day 0–2) that markedly diminished by day 8, indicative of effective bacterial elimination in vivo.

Physiological responses were further assessed through body weight and wound healing trajectories. All groups maintained stable weights (Figure S5c,g), confirming the systemic biosafety of the SERS probe and laser irradiation. Healing rate analyses (Figure S5d,h) revealed that the SERS probe significantly accelerated wound closure compared to all other treatments, in both BALB/c and db/db models. Importantly, the diabetic mice, known for impaired repair and chronic infection, also benefitted substantially, with outcomes comparable to or surpassing those in BALB/c mice. These findings indicate that the SERS probe not only exerts potent antibacterial activity but also promotes tissue regeneration, possibly by alleviating the inflammatory burden through effective infection control.

In contrast, the AuPB NP group displayed only limited therapeutic effects, suggesting that the plasmonic core alone is insufficient without the functional architecture of the SERS probe. To further validate bacterial clearance, CFU quantification and Raman signal analysis were performed. As shown in Figure S3e,i, CFU counts of both *E. coli* and *P. aeruginosa* were significantly reduced in the SERS probe group compared to all other groups, with consistent trends across both mouse models. Quantitative Raman analysis (Figure S5f,j) showed strong initial signals (day 0–2) followed by a sharp decline by day 8 in the SERS probe group, aligning with the CFU data. Bright‐field overlays of SERS maps (Figure S6) further supported these findings. Collectively, these results demonstrate that the SERS probe not only achieves therapeutic efficacy against complex infections but also allows real‐time, non‐invasive monitoring of infection dynamics in vivo. It's combined antibacterial and diagnostic capabilities position it as a promising theranostic platform for managing polymicrobial infections, especially in vulnerable populations such as diabetic patients. Overall, this study highlights the translational potential of SERS‐based theranostics as a next‐generation antimicrobial strategies in wound care.

### 2.7 Mechanism of Promoting Wound Healing

To further elucidate the mechanism by which the SERS probe promotes wound healing in diabetic wounds complicated by bacterial infection, we conducted immunofluorescence staining to analyze macrophage polarization. Wounds on db/db mice were infected with *E. coli*, *P. aeruginosa*, or a mixture of both pathogens, and then treated with PS, AuPB NPs, CS, or SERS probes under laser irradiation. Tissue samples were collected at days 2 and 8 post‐treatment for immunostaining of CD86 (green, marking pro‐inflammatory M1 macrophages) and CD206 (red, marking anti‐inflammatory M2 macrophages) [38]. As shown in Figure 8a–d, wounds treated with PS or AuPB NPs exhibited intense CD86 expression with minimal CD206 staining on day 2, reflecting persistent inflammatory response and insufficient resolution. In contrast, the SERS probe group showed lower CD86 and markedly elevated CD206 expression by day 8, indicating an effective transition of macrophages from an M1‐dominated to an M2‐dominated state, which is critical for wound remodeling and tissue repair. The CS group exhibited moderate modulation of polarization but did not reach the level observed in the SERS probe‐treated group.

**FIGURE 8:**
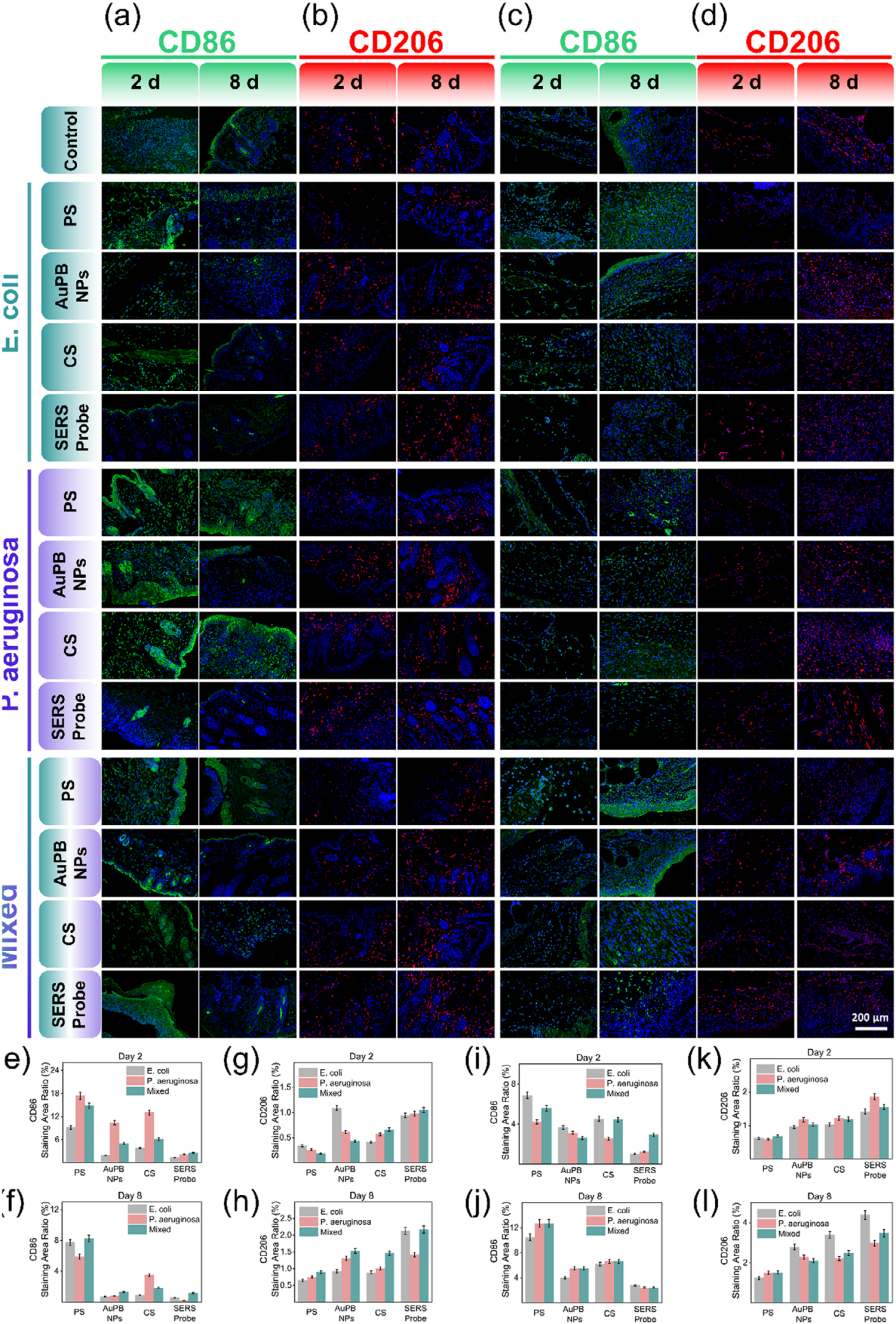
SERS probes modulate macrophage polarization to promote wound healing in bacterial‐infected diabetic wounds. Macrophage polarization immunofluorescence analysis conducted on db/db diabetic mice wounds infected with *E. coli*, *P. aeruginosa*, or mixed bacterial infections under different treatment strategies, with observations at day 2 and day 8. (a, c) Experimental groups includes PS, AuPB NPs, CS, and SERS probe treatments. (b, d) CD86 (green) staining marks M1 macrophages; (e‐l) CD206 (red) staining marks M2 macrophages. Quantitative analysis of the CD86 and CD206 staining area ratios corresponding to (a‐d). Scale bar = 200 µm.

Quantitative analysis of the stained tissue sections revealed dynamic changes in macrophage polarization across different treatment groups (Figure 8e–l). On day 2 (Figure 8e,g,i), CD86+ M1 macrophages were dominant in all infected wounds, particularly in the PS and AuPB NPs groups, consistent with the acute inflammatory phase. However, in the SERS probe group, the proportion of CD86+ cells were significantly reduced compared to other groups, even at this early stage, suggesting accelerated inflammation resolution. Concurrently, CD206+ M2 macrophages were notably more abundant in the SERS probe group, indicating early initiation of a pro‐healing phenotype (Figure 8g,i). By day 8 (Figure 8f,h,j,l), polarization differences became more pronounced. CD86+ cell ratios declined across all groups but remained elevated in the PS and AuPB NP groups. In contrast, the SERS probe group displayed the lowest CD86 and highest CD206 ratios, confirming a robust shift toward a regenerative macrophage profile. These trends were observed consistently across *E. coli*, *P. aeruginosa*, and mixed infections, although the mixed‐infection wounds exhibited the highest inflammatory burden and showed the most pronounced benefit from SERS probe treatment.

The ability of SERS probes to modulate macrophage polarization in infected diabetic wounds suggests a multifaceted therapeutic mechanism extending beyond direct bacterial eradication. By suppressing M1‐mediated inflammation early and promoting M2 upregulation, the SERS probe fosters an immunologically favorable environment for tissue regeneration. This function is especially critical in diabetic wounds, where chronic inflammation and impaired macrophage plasticity severely hinder healing. The probe's photodynamically activated antibacterial action likely plays a dual role: first, by directly reducing the bacterial load and infection‐induced inflammation; and second, by modulating the local microenvironment through reduced oxidative stress and cytokine normalization, thus promoting M2 polarization. Compared to conventional antibiotics (CS) or passive nanomaterials (AuPB NPs), SERS probes provide a smart, responsive, and biointeractive platform for advanced wound care. These findings not only reinforce the therapeutic potential of SERS probes but also highlight the importance of immune modulation as a critical target in chronic wound management.

### 2.8 Flow Cytometry for Evaluation of Macrophage Polarization

Flow cytometry analysis further performed to verify the in vitro anti‐inflammatory and macrophage‐polarization‐regulating effects of the SERS probe. CD86 and CD206 were selected as the representative markers of pro‐inflammatory M1 and anti‐inflammatory M2 macrophages, respectively. As shown in Figure 9a,c and d, in the E. coli‐inflammatory model, the control, laser irradiation, CS, AuPB NPs, and SERS probe groups exhibited relatively high CD86 fluorescence intensity and weak CD206 expression, indicating the pro‐inflammatory macrophage phenotype. In contrast, the SERS probe with laser irradiation group significantly increased CD206 expression while maintaining a relatively low CD86 levels. In addition, the quantitative analysis exhibited that CD206 fluorescence intensity in the SERS probe with laser irradiation group was relatively higher than that in the control, laser irradiation, CS, AuPB NPs, and SERS probe alone groups. Consistently, the CD86/CD206 fluorescence intensity ratio decreased remarkably after SERS probe with laser irradiation treatment, which suggested that the combined PDT‐antibiotic strategy efficiently promoted macrophage polarization toward anti‐inflammatory M2 phenotype. A similar regulatory trend was observed in the P. aeruginosa inflammatory model. Flow cytometry results showed that SERS probe with laser irradiation increased the population of CD206‐positive cells compared with the control and single‐treatment groups (Figure 9b). Quantitative fluorescence intensity analysis further confirmed that CD206 expression remained higher that CD86 expression across treatment groups, and the SERS probe with laser irradiation group showed enhanced CD206 fluorescence intensity compared with the most control treatments (Figure 9e). Although P. aeruginosa stimulation induced a stronger inflammatory response than E. coli, as reflected by the relatively increased CD86 signal in some treatment groups, the SERS probe with laser irradiation group maintained a CD206‐dominant phenotype, which indicated that the treatment did not aggravate inflammatory macrophage activation but induced anti‐inflammatory macrophage state. Accordingly, these flow cytometry results demonstrated that the SERS probe with laser irradiation treatment could regulate macrophage polarization in vitro, especially by enhancing CD206‐related M2 polarization and improving the CD86/CD206 balance. This anti‐inflammatory effect may be attributed to efficient bacterial elimination by the combined effect of PDT‐CS treatment, which reduces bacteria‐induced inflammatory stimulation and creates a favorable microenvironment for inflammation resolution and tissue repair.

**FIGURE 9:**
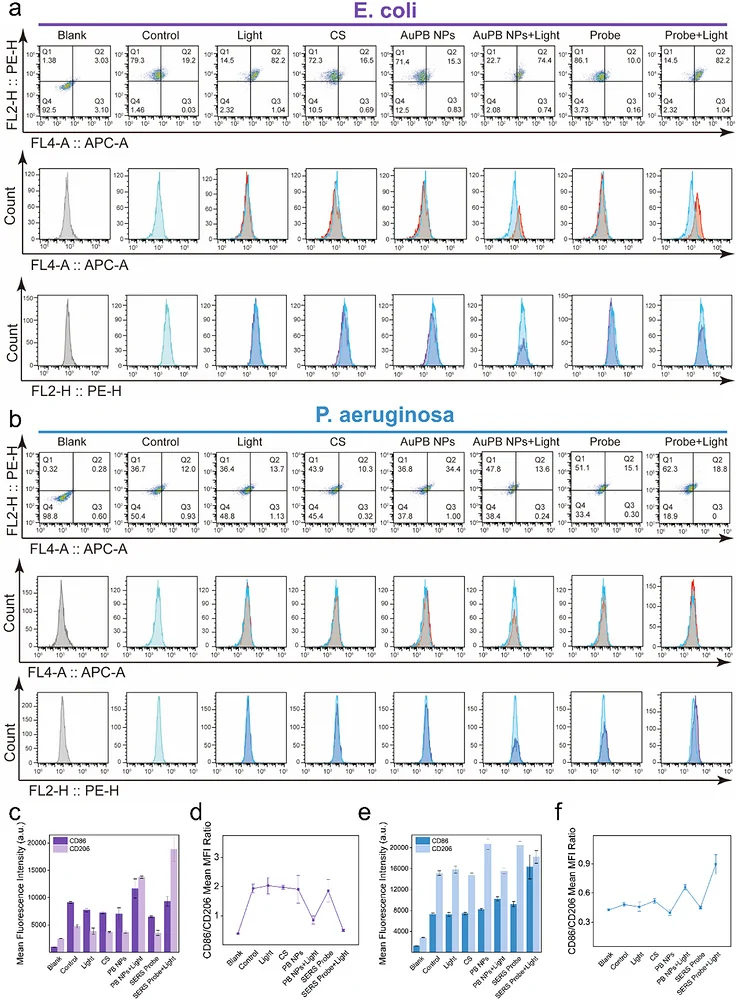
Flow cytometry analysis results of macrophages under different treatment conditions. In RAW264.7 macrophage models infected with (a) E. coli and (b) P. aeruginosa, flow cytometry analysis was performed under different treatment conditions including Blank, Control, Light, CS, AuPB NPs, AuPB NPs + Light, SERS probe, and SERS probe + Light. The top row was the scatter plot of the FL2‐H (PE‐H) channel; the middle and bottom rows showed histograms of fluorescence intensity distribution in the FL4‐A::APC‐A and FL2‐H::PE‐H channels for each treatment group compared to the control group, respectively. (c, e) Quantitative analysis of median fluorescence intensity (MFI) in the APC‐A and PE‐H channels under E. coli and P. aeruginosa infection conditions, respectively. (d, f) The median fluorescence intensity ratio of CD86/CD206 for E. coli and P. aeruginosa, respectively.

### 2.9 Histological and Immunohistochemical Evaluation

To evaluate the pro‐healing capability of the SERS probe in bacterially infected wounds, BALB/c mice were infected with *E. coli*, *P. aeruginosa*, or a mixed bacterial suspension and treated with different regimens: PS, AuPB NPs, CS, or SERS probes activated with 650 nm laser irradiation. Tissue samples were harvested at days 2 and 8 post‐treatment and subjected to histological and immunohistochemical analyses to assess tissue regeneration, extracellular matrix reconstruction, and angiogenesis. Hematoxylin and eosin (HE) staining (Figure 10a) revealed that the SERS probe group better preserved tissue architecture with notably reduced inflammatory cell infiltration, particularly by day 8, across all infection types. Masson's trichrome staining (Figure 10b) further demonstrated significant collagen deposition in SERS probe‐treated wounds, with dense and well‐organized, blue‐stained collagen bundles, in contrast to sparse and irregular patterns observed in the PS or AuPB NP groups. The more detailed H&E and Masson staining images were exhibited in Figures S8 and S9. Quantitative analysis confirmed these results, showing the highest collagen fiber area ratios in the SERS group at both time points (Figure 10f, g), indicating accelerated wound matrix reconstruction, a key step in scar tissue maturation.

**FIGURE 10:**
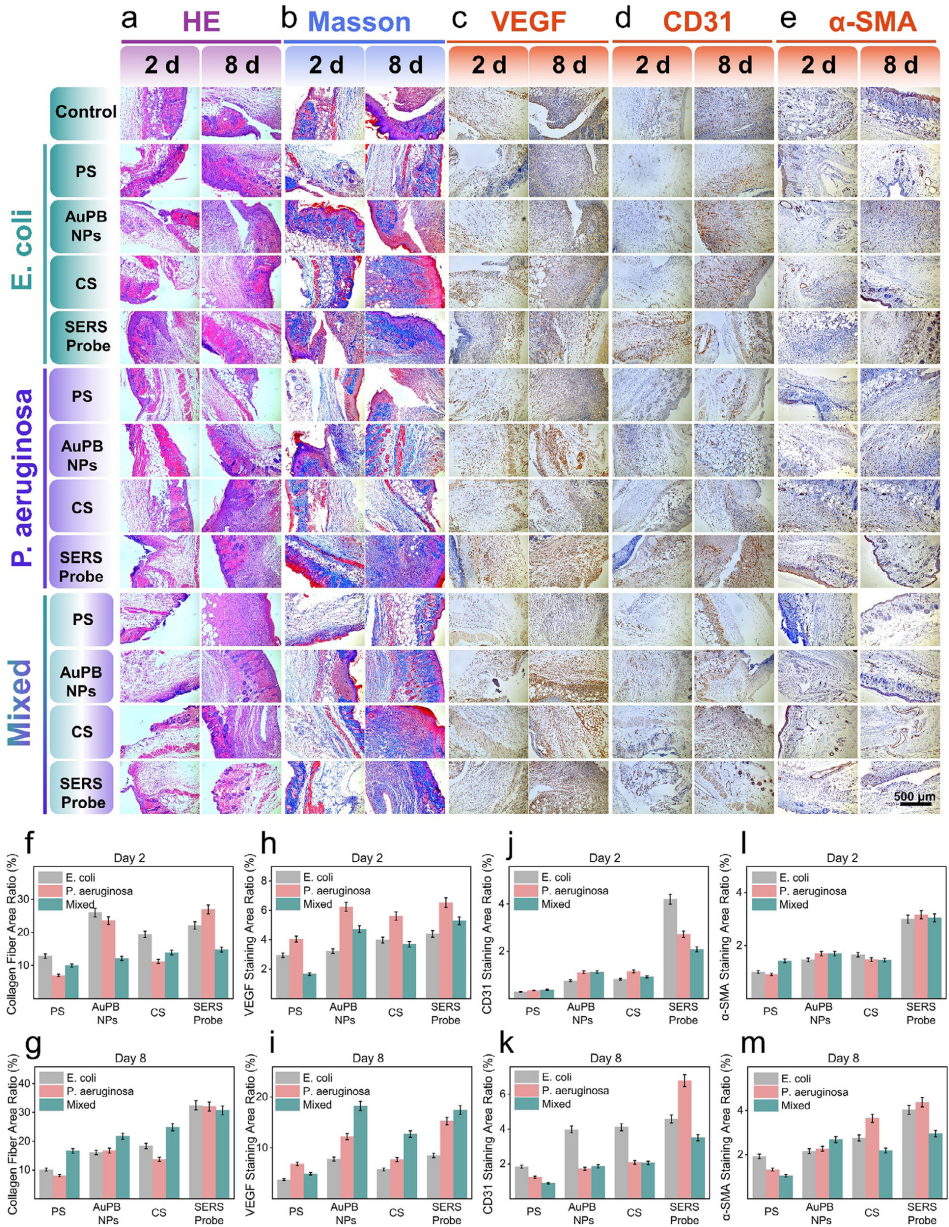
Wound healing in infected lesions by enhancing angiogenesis and collagen deposition with SERS probes. BALB/c mouse models infected with *E. coli*, *P. aeruginosa*, or mixed bacterial infections were utilized, followed by different treatments including PS, AuPB NPs, CS, and SERS probe administration. Histological and immunohistochemical analyses were performed to assess wound healing under different therapeutic strategies at days 2 and 8. (a) HE staining for evaluating tissue structural changes. (b) Masson's trichrome staining for visualizing collagen fiber deposition. (c) VEGF immunostaining for analyzing angiogenesis. (d) CD31 immunostaining for detecting endothelial cell markers. (e) α‐SMA immunostaining for assessing vascular smooth muscle cell activation. (f‐m) Quantitative analysis of staining area ratios corresponding to (a‐e). Scale bar = 500 µm.

Angiogenesis was evaluated using immunohistochemical staining for VEGF (angiogenic factor), CD31 (endothelial cell marker), and α‐SMA (vascular smooth muscle cell and myofibroblast marker) [39, 40]. As shown in Figure 10c–e, the SERS probe group exhibited markedly enhanced VEGF and CD31 expression compared with other treatments, suggesting robust angiogenic signaling and neovascularization. α‐SMA expression was also significantly elevated, indicating greater vascular remodeling and increased myofibroblast‐mediated wound contraction. These effects were most pronounced in mixed‐infection wounds, which typically exhibit the most severe healing impairment.

Quantitative evaluation (Figure 10f–m) supported these observations, with the SERS probe group demonstrating the highest staining area ratios for VEGF, CD31, and α‐SMA across all infection models and time points. Although CS treatment provided moderate benefits, it was consistently outperformed by SERS probe therapy, underscoring the added benefits of photodynamically activated treatment. Together, these findings suggest that the SERS probe not only eradicates infection and modulates immune responses but also actively promotes collagen matrix formation and vascular regeneration. This multifunctional capability positions the SERS probe as a next‐generation therapeutic for complex and chronic wound infections.

To assess the regenerative efficacy of the SERS probe in diabetic wounds with bacterial infections, db/db mouse models were established using *E. coli*, *P. aeruginosa*, or mixed bacterial inoculation. The mice were treated with PS, AuPB NPs, CS, or SERS probes combined with laser irradiation. On days 2 and 8 post‐treatment, wound tissues were harvested and subjected to histological and immunohistochemical analysis. HE staining (Figure S7a) revealed that wounds treated with the SERS probe exhibited improved epithelial integrity, reduced necrosis, and more organized dermal architecture, especially by day 8. Masson's trichrome staining (Figure S7b) demonstrated increased collagen fiber density and uniformity in the SERS group compared to other treatment groups. Quantitative collagen fiber area ratios (Figure S7f,g) confirmed that SERS probes significantly enhanced matrix remodeling at both time points across all infection models. Notably, wounds with mixed infections, despite showing the greatest initial impairment, exhibited the most pronounced recovery following SERS probe treatment.

Angiogenesis was evaluated via immunostaining of VEGF (vascular endothelial growth factor), CD31 (endothelial cell marker), and α‐SMA (smooth muscle actin), as shown in Figure S7c–e. The SERS group showed marked upregulation of VEGF and CD31, indicating robust angiogenic signaling and endothelial activation. Furthermore, α‐SMA staining revealed enhanced activation of vascular smooth muscle cells and myofibroblasts, key components in vessel stabilization and wound contraction. These effects were most notable in the mixed infection wounds, which typically exhibit impaired angiogenesis due to chronic inflammation. Quantitative analysis of staining areas (Figure S7h–m) demonstrated that the SERS probe group consistently achieved the highest VEGF, CD31, and α‐SMA staining ratios across all infection models at both time points. While CS treatment produced moderate improvements, and AuPB NPs led to minimal benefit, the SERS probe significantly outperformed both, demonstrating its dual functionality as an antimicrobial and pro‐regenerative properties. In conclusion, the SERS probe not only eradicates bacterial infection but also reprograms the wound microenvironment to favor matrix reconstruction and angiogenesis, highlighting a its potential as a powerful therapeutic strategy for chronic, infected diabetic wounds.

### 2.10 Clinical Sample Application

Furthermore, to evaluate the clinical applicability of the proposed SERS probe, we applied it to detect actual patient wound samples and to access its diagnostic accuracy as well as its potential for guiding infection management. As shown in Figure 11a, the workflow illustrates the standardized procedure for SERS‐based clinical analysis, including collection of infected wound tissue samples from patients, incubation with the SERS probe, and SERS signal detection with 633 nm laser excitation. A total of 36 clinical samples were analyzed, including 21 cases of Gram‐positive bacteria and 15 cases of Gram‐negative bacteria infection. Figure 11b presents bright‐field images and corresponding SERS mapping results, which clearly demonstrate significant differences in Raman signal distribution. Gram‐positive infection samples exhibited relatively lower SERS intensity, whereas Gram‐negative infection generated much stronger SERS signals in the Raman‐silent region, confirming that the probe can directly detect bacterial presence in clinically complex environment.

**FIGURE 11:**
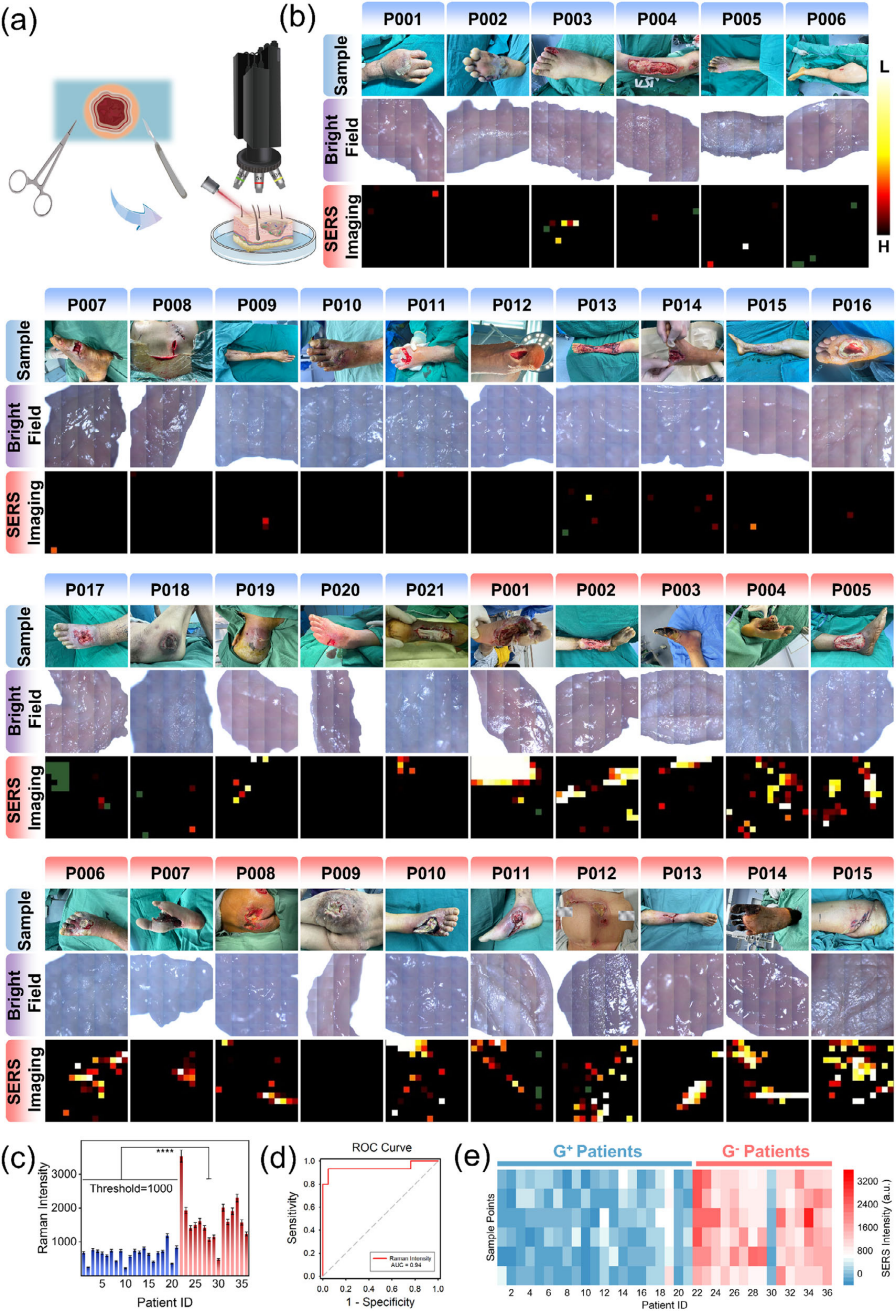
SERS probes for clinical sample monitoring. (a) Schematic illustration of the detection process. (b) SERS imaging of 21 clinical wound samples infected with Gram‐positive bacteria and 15 clinical wound samples infected with Gram‐negative bacteria. (c) Comparison of SERS signal intensity for each clinical sample. (d) ROC analysis of 21 Gram‐positive bacteria‐infected patients and 15 Gram‐negative bacteria‐infected patients detected by SERS probe. AUC: area under the curve. (e) Analysis of heatmap of 36 bacteria‐infected patients. Error bars represent the standard error (n = 3).

Quantitative analysis of SERS signals further validated the probe's diagnostic performance. The obtained SERS results showed strong concordance with those from the gold standard method (Table S1). As illustrated in Figure 11c, the mean SERS signal intensity for each sample was quantified, and the threshold value of 1000 a.u. was established to distinguish between background noise and clinically relevant signals. Receiver operating characteristic (ROC) analysis demonstrated excellent diagnostic performance of the SERS probe with an area under the curve (AUC) of 0.94, indicating excellent sensitivity and specificity for distinguishing Gram‐positive and Gram‐negative infections (Figure 11d). Furthermore, heatmap visualization of SERS signal distributions across all patient samples revealed consistent signatures for Gram‐negative infections, while Gram‐positive cases displayed weaker but still distinguishable SERS patterns (Figure 11e). Collectively, these results demonstrate that the proposed SERS probe enables dynamic, reliable, and accurate classification of bacterial type and infection severity in patient‐derived clinical samples.

## 3 Conclusion

Bacterial wound infection remains one of the clinical challenges in both acute and chronic wound care, primarily due to biofilm formation, multidrug resistance and persistent inflammation that delay tissue repair and increase the risk of recurrence. Conventional antibiotic therapy alone often provides insufficient, since high dose administration not only increases systemic toxicity but also accelerates resistance development. Moreover, currently available diagnostic approaches, such as culture or PCR, are time‐consuming, invasive, or incapable of distinguishing viable from dead bacteria.

Here we report a multifunctional Raman‐silent‐region SERS probe that integrates PB‐mediated PDT, CS‐based antibiotic delivery, and real‐time infection monitoring. The proposed platform differs substantially from conventional theranostic systems that typically depend on exogenous Raman tags or single‐modality antibacterial strategies. In contrast, it exploits the intrinsic silent‐region Raman signal of PB for simultaneous interference‐free bacterial detection and photodynamically induced antibacterial action. This dual role of PB functioning both as photosensitizer and Raman reporter, directly addresses long‐standing challenges in achieving concurrent bacteria killing and monitoring in infected wounds.

The combined antibacterial mechanism of PB‐mediated PDT and CS release proved critical in overcoming the limitations of monotherapy. Under near‐infrared irradiation, the PB shell efficiently generated singlet oxygen, which disrupted bacterial membranes and damaged intracellular components. Meanwhile, CS selectively bound to the outer membrane of Gram‐negative bacteria, thereby enhancing permeability and promoting cell lysis. This combined mechanism achieved > 90% reduction of *E. coli* and *P. aeruginosa* populations, achieved with significantly reduced antibiotic dosages, thus lowering the risk of resistance development. In vivo, these outcomes translated into accelerated wound closure in both BALB/c and diabetic models, accompanied by reduced bacterial burden, enhanced M2 macrophage polarization, angiogenesis and collagen deposition. More importantly, silent‐region SERS imaging enabled real‐time, non‐invasive monitoring of bacterial load for evaluation of therapeutic efficiency. This strategy achieved robust and interference‐free signals to discriminate Gram‐positive and Gram‐negative infections. Clinical validation using 36 patient samples further confirmed its diagnostic robustness, yielding an AUC of 0.94.

The SERS probe also demonstrated an excellent biosafety prfile supported by stable body weights, normal serum biochemistry, and lack of histopathological toxicity in treated animals. Although CS was chosen for Gram‐negative coverage, incorporation of alternative or broad‐spectrum antibiotics could really extend the platform to mixed bacterial infections. Likewise, optimization of PB shell properties could further enhance ROS yield, drug release dynamics, and systemic biodistribution. Nevertheless, there are still some challenges. Validations in large animal models is necessary to fully recapitulate the pathophysiological complexity of human wounds. Meanwhile, systematic studies on long‐term biodistribution, clearance, and repeated dosing safety are also warranted. Beyond wound infections, the design principles demonstrated here, integrating silent‐region Raman diagnostics with multifunctional therapy, could be broadly extended to address implant‐related infections, osteomyelitis, or even respiratory diseases.

In conclusion, we developed a multifunctional Raman‐silent SERS probe that combines photodynamic therapy, antibiotic release, and real‐time bacterial monitoring for effective management of infected wounds. Utilizing Prussian blue not only as a Raman reporter in the spectroscopically silent region but also as a photosensitizer for ROS generation, the probe enables precise, background‐free SERS detection alongside combined antibacterial activity when combined with CS. Comprehensive in vitro and in vivo studies, including diabetic wound models, verified robust bacterial clearance, accelerated tissue repair, immunomodulation via macrophage polarization, and enhanced angiogenesis and collagen deposition. Furthermore, clinical testing confirmed its capability to accurately discriminate Gram‐positive and Gram‐negative infections. This integrated theranostic platform offers a promising and translationally relevant strategy for managing complex bacterial infections and promoting wound regeneration.

## 4 Experimental Section

### 4.1 Materials

Gold (III) chloride trihydrate (HAuCl_4_·3H_2_O) was obtained from Civic Chemical Co., Ltd., and trisodium citrate was from HopeLink (Xilong Scientific). Colistin sulfate (CS), Prussian blue (PB), potassium ferricyanide (K_3_[Fe(CN)_6_]), potassium ferrocyanide (K_4_[Fe(CN)_6_]), ferric chloride (FeCl_3_) and colistin sulfate were purchased from Aladdin. Escherichia coli (E. coli, ATCC 25922) and Pseudomonas aeruginosa (P. aeruginosa, ATCC 27853) were obtained from the Guangdong Microbial Culture Collection Center (GDMCC). Human immortalized keratinocytes (HaCaT) and mouse embryonic fibroblast cells (NIH‐3T3) were purchased from Wuhan Procell Life Science & Technology Co., Ltd., while human umbilical vein endothelial cells (HUVECs) were obtained from CytoNiche Biotechnology Co., Ltd. Female BALB/c and db/db mice were purchased from Tianqin Biotechnology (Changsha, China) and acclimatized for one week under a 12 h light/12 h dark cycle in a specific pathogen‐free (SPF) environment.

### 4.2 Instruments

UV–vis spectra and optical density (OD) measurements were recorded using a SYNERGY H1 microplate reader. The photodynamic therapy system utilized a 650 nm xenon lamp (PE300‐T8.8, Beijing CEAuLight Co.). A NanoDrop One spectrophotometer was used to quantify nucleic acid leakage. Nanoparticle size and zeta potential were measured by a nanoparticle analyzer (SZ‐100 V2, HORIBA Scientific). Bacterial viability was assessed by confocal laser scanning microscopy (CLSM, Olympus FV3000). Morphology and ultrastructure of bacteria and nanomaterials were visualized using transmission electron microscopy (TEM, HITACHI HT7800) and scanning electron microscopy (SEM, HITACHI SU8100).

### 4.3 Synthesis of Nanomaterials

Gold nanoparticles (Au NPs) were synthesized using a seed‐mediated growth method [41]. Briefly, 48.5 mg of trisodium citrate was dissolved in 75 mL of ultrapure water to prepare a 2.2 mM citrate solution. Under continuous heating and stirring, 0.5 mL of 25 mM HAuCl_4_ was added once the solution reached boiling. After boiling for 15 min, the temperature was stabilized at 90 °C, followed by 0.5 mL of 60 mM trisodium citrate. 2 min later, HAuCl_4_ was added and this step was repeated for 12 cycles. After cooling to room temperature, the Au NPs solution was stored at 4 °C.

Next, 0.5 mM K_3_[Fe(CN)_6_], 0.1 mM K_4_[Fe(CN)_6_], and FeCl_3_ were prepared. In 5 mL of Au NPs solution, 0.5 mL of K_3_[Fe(CN)_6_] was slowly added and stirred for 5 min, followed by dropwise addition of 0.5 mL of the PB precursor solution (containing K_4_[Fe(CN)_6_] and FeCl_3_) at 2 mL/h. The reaction was maintained for 5 h under stirring to yield AuPB NPs [26, 42, 43].

The AuPB NPs were redispersed in Na_2_CO_3_ buffer (pH = 9), followed by addition of 10 µL of 1 mg/mL colistin sulfate per 1 mL of AuPB NPs solution [43]. The mixture was stirred for 2 h, then blocked with 10 µL of 10% BSA for 1 h. The particles were washed and redispersed in ultrapure water three times and stored at 4 °C as the final SERS probes [44].

### 4.4 SERS Detection of Bacteria

A series of E. coli and P. aeruginosa suspensions were prepared at concentrations ranging from 1×10^3^ CFU/mL to 1×10^7^ CFU/mL. Equal volumes of bacterial suspensions were reacted with the SERS probe for 30 min each. Afterward, unbound bacteria and probe were separated by differential centrifugation. The precipitate of the bacterial‐SERS probe complex was collected, resuspended in PBS buffer, and subjected to Raman spectroscopy. The characteristic Raman peak at 2133 cm^−1^ was recorded, and its relationship with the logarithm of bacterial concentration was analyzed.

The SERS probe solution was centrifuged and resuspended in physiological saline, 6.1 mM glucose solution, and 14 mM glucose solution, respectively. The spectral signals were measured, and the changes in the characteristic Raman peak at 2133 cm^−1^ under different conditions were compared.

### 4.5 Photothermal Test of SERS Probe

Equal volumes of SERS probe, AuPB NPs, and PBS buffer solution were collected in EP tubes for photothermal performance testing. The tubes were continuously irradiated with 650 nm near‐infrared light for 5 min (200 mW/cm^2^), and the temperature changes within 5 min (0, 30, 60, 120, 180, 240, 300 s) were monitored using an infrared thermal imager.

### 4.6 Photodynamic Properties

A 1 mM stock solution of singlet oxygen sensor green (SOSG) was prepared by dissolving 100 µg of SOSG in 165 µL methanol. A 20 µM working solution was prepared in PBS. The SERS probe solution was mixed with SOSG working solution to a final concentration of 1 µM. Samples were irradiated with a 650 nm laser (200 mW/cm^2^) for 0, 1, 2, 5, 10, 15, and 20 min, and the fluorescence spectra (λ_ex_ = 488 nm, λ_em_ = 500–700 nm) were recorded using a microplate reader.

### 4.7 Biofilm Tests

100 µL of 1 × 10^7^ CFU/mL fresh P. aeruginosa suspension and 900 µL of LB broth were added to 24‐well plates and incubated at 37°C for 20 h to form the stable biofilm. After biofilm formation, different treatments were applied, including PBS, light irradiation, AuPB NPs + light irradiation, CS, SERS probe, and SERS probe + light irradiation, followed by incubation for 30 min. The light irradiation group was treated with 650 nm at 200 mW/cm^2^ near‐infrared laser irradiation for 5 min. The incubation was then carried out at 37°C for 24 h. The supernatant was carefully aspirated, and the plates were washed with PBS for three times to remove free bacteria. SYTO 9 staining solution and an appropriate amount of PBS were added, and the plates were incubated in the dark for 10 min. After discarding the staining solution, sterile PBS was added, and confocal fluorescence imaging was performed to observe the biofilm, acquiring 3D images and recording changes in biofilm thickness. The biofilm thickness of different treatment groups was analyzed and quantified.

100 µL of 1 × 10^7^ CFU/mL fresh P. aeruginosa suspension was added to a 24‐well plate and subjected to different treatments, including PBS, light irradiation, AuPB NPs + light irradiation, CS, and SERS probe + light irradiation. LB broth was then added, and the plates were incubated at 37 °C for 24 h (the control group was blank LB broth). The light irradiation groups were treated with a 650 nm laser at 200 mW/cm^2^ for 5 min. The supernatant LB broth was then discarded, and the plates were washed three times with PBS buffer. After complete drying, the plates were fixed with 4% paraformaldehyde for 40 min, followed by thorough drying. 0.01% crystal violet staining solution was added for 15 min, followed by washing and drying. The plates were then destained with 30% acetic acid solution in a shaker at 37°C. After destaining, 100 µL of the solution was used to measure OD590.

### 4.8 Colistin Loading Efficiency

Colistin solutions of varying concentrations (1–200 µg/mL) were used to establish a standard curve via a BCA protein assay. The encapsulation efficiency was calculated from the residual colistin concentration in the supernatant after centrifugation of the SERS probe solution.

### 4.9 Bacterial Targeting Capability

100 µL of bacterial suspension (1×10^7^ CFU/mL) was added to sterile glass slides and incubated at 37°C for 20 min. After washing with PS, AuPB NPs, CS or SERS probes were added and co‐incubated for 0, 5, 15, 30, or 60 min. Unbound nanoparticles were removed with PBS, and bacterial targeting was observed via confocal Raman imaging.

### 4.10 In Vitro Antibacterial Activity

The minimum inhibitory concentration (MIC) of SERS probes against *E. coli* and *P. aeruginosa* was determined by incubating bacteria with different concentrations of SERS probes (1.5–0.094 nM) in LB medium, with or without laser irradiation (650 nm, 200 mW/cm^2^, 5 min). After 24 h incubation, OD_600_ was measured.

The combined antibacterial effects were evaluated using five groups: control, PS, AuPB NPs, colistin sulfate (CS), and SERS probe, with or without laser irradiation. Treated bacterial solutions were diluted 10^4^‐fold, plated on LB agar, and incubated to count CFUs and calculate bacterial survival.

Live/dead staining was performed using SYTO 9 (λ_ex_ = 488 nm, λ_em_ = 510–550 nm) and propidium iodide (PI, λ_ex_ = 561 nm, λ_em_ = 617–667 nm) followed by CLSM observation.

### 4.11 Antibacterial Mechanism Studies

To identify ROS types, DCFH‐DA, SOSG, or DHE were added to bacteria treated with SERS probes or controls and incubated for 30 min [45]. Fluorescence was then imaged by CLSM. Bacterial morphology after treatment with PS, AuPB NPs, CS, or SERS probes was observed by TEM and SEM after fixation. To assess membrane potential, DiSC_3_(5) dye (0.8 mM) was added to bacterial suspensions in black 96‐well plates, and the fluorescence (Ex/Em = 620/670 nm) was measured before and after treatments including PBS, HEPES buffer, 0.5% Triton X‐100, SERS probe, and SERS probe + laser irradiation. Nucleic acid leakage was quantified from supernatants using a NanoDrop one spectrophotometer (OD_260_), following different treatments.

### 4.12 In Vivo Antibacterial and SERS Imaging

All animal procedures were approved by the Animal Ethics Committee of Hainan Medical University (HY2025‐24) and the Human Ethics Committee of Hainan Medical University (HY2025‐13). BALB/c mice were randomly divided into groups infected with *E. coli*, *P. aeruginosa*, or co‐infected, and treated with PS, AuPB NPs, CS, or SERS probes (n = 3), including control groups. Full‐thickness wounds (5 mm) were made on dorsal skin using a sterile biopsy punch, followed by inoculation with 30 µL of bacterial suspension or saline.

On day 0, treatments were applied and irradiated with the laser (if applicable). Wound healing progression was documented photographically and via SERS imaging on days 2, 4, 6, and 8. On day 8, mice were euthanized at the end of the experiment and wound tissues were harvested for CFU count, H&E and Masson's staining, IHC (VEGF, CD31, α‐SMA), and IF (CD86, CD206). Furthermore, wound modeling in diabetic db/db mice followed similar procedures.

### 4.13 Biocompatibility Assessment

Cytotoxicity was assessed via a CCK‐8 assay on HaCaT, NIH‐3T3, and HUVEC cells [46]. Cells (1×10^4^ cells/well) were incubated with varying SERS probe concentrations for 24 h, followed by 2 h incubation with CCK‐8 solution. Absorbance at 450 nm was measured to calculate viability. Hemolysis was evaluated using BALB/c mouse erythrocytes. Red blood cells were mixed with PBS (negative control), 1% Triton X‐100 (positive control), or SERS probe solutions (0.0625–1.0 nM), and incubated at 37 °C for 4 h. After centrifugation, the absorbance at 540 nm was measured to determine hemolysis rate.

### 4.14 Clinical Sample Analysis

Clinically infected skin tissues were obtained from the patients in the Wound Repair Department, the First Affiliated Hospital of Hainan Medical University, under ethical approval. Samples were immediately imaged via SERS and stored at ‐80 °C. Inclusion criteria: (1) no age or gender restriction; (2) confirmed bacterial infection; (3) clearly defined wound site; (4) no antibiotic use within or withdrawn ≥24 h before sampling. Exclusion criteria: (1) necrotic wounds; (2) prior antibacterial treatment affecting sampling; (3) insufficient or contaminated tissue.

### 4.15 Statistical Analysis

All data were analyzed using Origin 2024, and SPSS 25.0. Raman intensity analysis of SERS imaging was conducted using Image J. Results are presented as mean ± standard deviation (SD). One‐way ANOVA was used for group comparisons; *P* < 0.05 was considered statistically significant (*: < 0.05, **: < 0.01, ***: < 0.001, ****: < 0.0001).

## Author Contributions

S.C. and R.W. conceived and designed the study. H.D. performed the probe synthesis and in vitro experiments. H.D., D.Z. and W.Z. performed in vivo experiments, histology experiments and cell imaging experiments. F.Y. and J.C. analyzed the data. R.W. and J.C. drafted the manuscript. All authors discussed the results and commented on the manuscript.

## Conflicts of Interest

The authors declare no conflict of interest.

## Supporting information




**Supporting File**: advs76574‐sup‐0001‐SuppMat.docx.

## Data Availability

The data that support the findings of this study are available from the corresponding author upon reasonable request.

## References

[1] A. Uberoi , A. McCready‐Vangi , and E. A. Grice , “The Wound Microbiota: Microbial Mechanisms of Impaired Wound Healing and Infection,” Nature Reviews Microbiology 22 (2024): 507–521. 10.1038/s41579-024-01035-z 38575708

[2] X. Ding , Q. Tang , Z. Xu , et al., “Challenges and Innovations in Treating Chronic and Acute Wound Infections: From Basic Science to Clinical Practice,” Burns & Trauma 10 (2022): tkac014. 10.1093/burnst/tkac014 PMC9123597 35611318

[3] V. Falanga , R. R. Isseroff , A. M. Soulika , et al., “Chronic wounds,” Nature Reviews Disease Primers 8 (2022): 50. 10.1038/s41572-022-00377-3 PMC10352385 35864102

[4] W. R. Miller and C. A. Arias , “ESKAPE Pathogens: Antimicrobial Resistance, Epidemiology, Clinical Impact and Therapeutics,” Nature Reviews Microbiology 22 (2024): 598–616. 10.1038/s41579-024-01054-w PMC13147291 38831030

[5] A. Bharadwaj , A. Rastogi , S. Pandey , S. Gupta , and J. S. Sohal , “Multidrug‐Resistant Bacteria: Their Mechanism of Action and Prophylaxis,” BioMed Research International 2022 (2022): 5419874. 10.1155/2022/5419874 PMC9467707 36105930

[6] N. Dheman , N. Mahoney , E. M. Cox , J. J. Farley , T. Amini , and M. L. Lanthier , “An Analysis of Antibacterial Drug Development Trends in the United States, 1980–2019,” Clinical Infectious Diseases 73 (2021): e4444–e4450. 10.1093/cid/ciaa859 32584952

[7] S. Roy , I. Hasan , and B. Guo , “Recent Advances in Nanoparticle‐Mediated Antibacterial Applications,” Coordination Chemistry Reviews 482 (2023): 215075.

[8] D. G. Armstrong , A. J. M. Boulton , and S. A. Bus , “Diabetic Foot Ulcers and Their Recurrence,” New England Journal of Medicine 376 (2017): 2367–2375. 10.1056/NEJMra1615439 28614678

[9] G. Jing , C. Hu , K. Fang , Y. Li , and L. Wang , “How Nanoparticles Help in Combating Chronic Wound Biofilms Infection?,” International Journal of Nanomedicine 19 (2024): 11883–11921. 10.2147/IJN.S484473 PMC11575445 39563901

[10] J. McDermott , L. S. Kao , J. A. Keeley , A. Grigorian , A. Neville , and C. de Virgilio , “Necrotizing Soft Tissue Infections,” JAMA Surgery 159 (2024): 1308. 10.1001/jamasurg.2024.3365 39259555

[11] B. A. Lipsky and C. Hoey , “Topical Antimicrobial Therapy for Treating Chronic Wounds,” Clinical Infectious Diseases 49 (2009): 1541–1549. 10.1086/644732 19842981

[12] F. Zhao , Y. Su , J. Wang , et al., “A Highly Efficacious Electrical Biofilm Treatment System for Combating Chronic Wound Bacterial Infections,” Advanced Materials 35 (2023): 2208069. 10.1002/adma.202208069 PMC9918715 36385439

[13] Y. Feng , C. C. Tonon , S. Ashraf , and T. Hasan , “Photodynamic and Antibiotic Therapy in Combination Against Bacterial Infections: Efficacy, Determinants, Mechanisms, and Future Perspectives,” Advanced Drug Delivery Reviews 177 (2021): 113941. 10.1016/j.addr.2021.113941 34419503

[14] W. Xiu , L. Wan , K. Yang , et al., “Potentiating Hypoxic Microenvironment for Antibiotic Activation by Photodynamic Therapy to Combat Bacterial Biofilm Infections,” Nature Communications 13 (2022): 3875. 10.1038/s41467-022-31479-x PMC9256606 35790729

[15] S. Benson , A. Kiang , C. Lochenie , et al., “Environmentally Sensitive Photosensitizers Enable Targeted Photodynamic Ablation of Gram‐Positive Antibiotic Resistant Bacteria,” Theranostics 13 (2023): 3814–3825. 10.7150/thno.84187 PMC10334829 37441588

[16] M. Piksa , C. Lian , I. C. Samuel , K. J. Pawlik , I. D. W. Samuel , and K. Matczyszyn , “The Role of The Light Source in Antimicrobial Photodynamic Therapy,” Chemical Society Reviews 52 (2023): 1697–1722. 10.1039/d0cs01051k 36779328

[17] J. Wang , X. Pan , X. Li , et al., “Photoimmunologic Therapy of Stubborn Biofilm via inhibiting Bacteria Revival and Preventing Reinfection,” Advanced Materials 37 (2025): 2411468. 10.1002/adma.202411468 39723739

[18] M. Yang , Y. Zhang , Z. Hou , et al., “NIR‐I Light‐Activated Antibiotic Delivery & PDT via TiO 2 /Graphene Metastructure for Enhanced Antibacterial Activity and Osseointegration of Ti Implants,” Advanced Healthcare Materials 14 (2025): 2500743. 10.1002/adhm.202500743 40145804

[19] L. Yang , S. Song , M. Yin , et al., “Antibiotic‐Based Small Molecular Micelles Combined with Photodynamic Therapy for Bacterial Infections,” Asian Journal of Pharmaceutical Sciences 18 (2023): 100810. 10.1016/j.ajps.2023.100810 PMC10236462 37274927

[20] C. S. Rossi , F. Coulon , S. Ma , Y. S. Zhang , and Z. Yang , “Microfluidics for Rapid Detection of Live Pathogens,” Advanced Functional Materials 33 (2023): 2212081.

[21] J. Yu , Y. Zheng , C. Song , and S. Chen , “New Insights Into the Roles of Fungi and Bacteria in the Development of medicinal plant,” Journal of Advanced Research 65 (2024): 137–152. 10.1016/j.jare.2023.12.007 PMC11518954 38092299

[22] A. Zhu , S. Ali , T. Jiao , Z. Wang , Q. Ouyang , and Q. Chen , “Advances in Surface‐Enhanced Raman Spectroscopy Technology for Detection of Foodborne Pathogens,” Comprehensive Reviews in Food Science and Food Safety 22 (2023): 1466–1494. 10.1111/1541-4337.13118 36856528

[23] X. Zhou , Z. Hu , D. Yang , et al., “Bacteria Detection: From Powerful SERS to Its Advanced Compatible Techniques,” Advanced Science 7 (2020): 2001739. 10.1002/advs.202001739 PMC7710000 33304748

[24] S. Lee , H. Dang , J. I. Moon , et al., “SERS‐Based Microdevices for Use as in Vitro Diagnostic Biosensors,” Chemical Society Reviews 53 (2024): 5394–5427. 10.1039/d3cs01055d 38597213

[25] Y. Sun , X. Zheng , H. Wang , et al., “Research Advances of Sers Analysis Method Based on Silent Region Molecules for Food Safety Detection,” Microchimica Acta 190 (2023): 387. 10.1007/s00604-023-05968-9 37700165

[26] W. Zhu , M. Gao , Q. Zhu , et al., “Monodispersed Plasmonic Prussian Blue Nanoparticles for Zero‐Background SERS/MRI‐Guided Phototherapy,” Nanoscale 12 (2020): 3292–3301. 10.1039/c9nr08471a 31971195

[27] Z. Li , X. Fan , Y. Liu , et al., “Engineering Mild‐Photothermal Responsive and NO Donor Prussian Blue Nanozymes Using Mild Synthesis for Inflammation Regulation and Bacterial Eradication in Periodontal Disease,” Advanced Materials 37 (2025): 2409840. 10.1002/adma.202409840 39690880

[28] A. Tong , C. Tong , J. Fan , et al., “Prussian Blue Nano‐Enzyme‐Assisted Photodynamic Therapy Effectively Eradicates MRSA Infection in Diabetic Mouse Skin Wounds,” Biomaterials Science 11 (2023): 6342–6356. 10.1039/d3bm01039b 37581536

[29] C. Zhang , Z. Xu , H. Di , E. Zeng , Y. Jiang , and D. Liu , “Gadolinium‐Doped Au@prussian Blue Nanoparticles as MR/SERS Bimodal Agents for Dendritic Cell Activating and Tracking,” Theranostics 10 (2020): 6061–6071. 10.7150/thno.42114 PMC7255006 32483438

[30] S. Zheng , J. Xiao , J. Zhang , et al., “Python‐Assisted Detection and Photothermal Inactivation of Salmonella Typhimurium and Staphylococcus Aureus on a Background‐Free SERS Chip,” Biosensors and Bioelectronics 247 (2024): 115913. 10.1016/j.bios.2023.115913 38091898

[31] N. G. Bastús , J. Comenge , and V. Puntes , “Kinetically Controlled Seeded Growth Synthesis of Citrate‐Stabilized Gold Nanoparticles of up to 200 nm: Size Focusing Versus Ostwald Ripening,” Langmuir 27 (2011): 11098. 10.1021/la201938u 21728302

[32] J. Han , H. D. Trinh , and S. Yoon , “Plasma‐Induced Nanogap Narrowing and Morphological Transformation in Gold Nanoparticle Assemblies,” Nanoscale 17 (2025): 972–981. 10.1039/d4nr03929g 39588608

[33] S. Kim , M. Fujitsuka , and T. Majima , “Photochemistry of Singlet Oxygen Sensor Green,” The Journal of Physical Chemistry B 117 (2013): 13985. 10.1021/jp406638g 24111566

[34] D. Yahav , L. Farbman , L. Leibovici , and M. Paul , “Colistin: New lessons on an old antibiotic,” Clinical Microbiology and Infection 18 (2012): 18–29. 10.1111/j.1469-0691.2011.03734.x 22168320

[35] M. Zhou , Y. Qian , J. Xie , et al., “Poly(2‐Oxazoline)‐Based Functional Peptide Mimics: Eradicating MRSA Infections and Persisters while Alleviating Antimicrobial Resistance,” Angewandte Chemie International Edition 59 (2020): 6412–6419. 10.1002/anie.202000505 32083767

[36] L. Gao , J. Cheng , Z. Shen , G. Zhang , S. Liu , and J. Hu , “Orchestrating Nitric Oxide and Carbon Monoxide Signaling Molecules for Synergistic Treatment of MRSA Infections,” Angewandte Chemie International Edition 61 (2022): 202112782. 10.1002/anie.202112782 34694047

[37] H. Elajaili , B. D. Lyttle , C. V. Lewis , et al., “Increased ROS and Persistent Pro‐Inflammatory Responses in a Diabetic Wound Healing Model (db/db): Implications for Delayed Wound Healing,” International Journal of Molecular Sciences 26 (2025): 4884. 10.3390/ijms26104884 PMC12112478 40430024

[38] Z. Feng , Q. Su , C. Zhang , et al., “Bioinspired Nanofibrous Glycopeptide Hydrogel Dressing for Accelerating Wound Healing: A Cytokine‐Free, M2‐Type Macrophage Polarization Approach,” Advanced Functional Materials 30 (2020): 2006454.

[39] L. Zong , R. Teng , H. Zhang , et al., “Ultrasound‐Responsive HBD Peptide Hydrogel with Antibiofilm Capability for Fast Diabetic Wound Healing,” Advanced Science 11 (2024): 2406022. 10.1002/advs.202406022 PMC11558141 39248340

[40] J. Hwang , K. L. Kiick , and M. O. Sullivan , “VEGF‐Encoding, Gene‐Activated Collagen‐Based Matrices Promote Blood Vessel Formation and Improved Wound Repair,” ACS Applied Materials & Interfaces 15 (2023): 16434–16447. 10.1021/acsami.2c23022 PMC10154048 36961242

[41] C. Qiu , W. Zhang , Y. Zhou , et al., “Highly Sensitive Surface‐Enhanced Raman Scattering (SERS) Imaging for Phenotypic Diagnosis and Therapeutic Evaluation of Breast Cancer,” Chemical Engineering Journal 459 (2023): 141502.

[42] Y. Guo , Y. Li , R. Fan , et al., “Silver@Prussian Blue Core–Satellite Nanostructures as Multimetal Ions Switch for Potent Zero‐Background SERS Bioimaging‐Guided Chronic Wound Healing,” Nano Letters 23 (2023): 8761. 10.1021/acs.nanolett.3c02857 37695577

[43] Y. Yin , Q. Li , S. Ma , et al., “Prussian Blue as a Highly Sensitive and Background‐Free Resonant Raman Reporter,” Analytical Chemistry 89 (2017): 1551–1557. 10.1021/acs.analchem.6b03521 28208262

[44] X. Qu , F. Yin , M. Pei , et al., “Modulation of Intratumoral Fusobacterium nucleatum to Enhance Sonodynamic Therapy for Colorectal Cancer with Reduced Phototoxic Skin Injury,” ACS Nano 17 (2023): 11466–11480. 10.1021/acsnano.3c01308 PMC10311605 37201179

[45] J. Peng , K. Du , J. Sun , et al., “Photocatalytic Generation of Hydrogen Radical (H·) with GSH for Photodynamic Therapy,” Angewandte Chemie International Edition 62 (2023): 202214991. 10.1002/anie.202214991 36537886

[46] Y. Yang , C. Zhang , Y. Cao , et al., “Bidirectional Regulation of I‐Type Lysozyme on Cutaneous Wound Healing,” Biomedicine & Pharmacotherapy 131 (2020): 110700. 10.1016/j.biopha.2020.110700 33152906

