# Supplementary material for: Dual‐Functional Silent‐Region SERS Nanoplatform for Real‐Time Bacterial Tracking and Wound Healing Therapy
Source: Adv Sci (Weinh). 2026 Jul 13;13(55):e76574. doi: 10.1002/advs.76574 (PMC13359399; doi:10.1002/advs.76574)
Supplement: Supplementary file 1 [file ADVS-13-e76574-s001.docx]

**Dual-Functional Silent-Region SERS Nanoplatform for Real-Time Bacterial Tracking and Wound Healing Therapy**

Hanbin Deng,^1, 2, +^, Rui Wang,^1, 2, +, *^ Dianqi Zhang,^1, 2, +^ Wei Zhang,^1, 2^ Jaebum Choo,^3, *^ Fabiao Yu,^1, 2, *^ Shaowen Cheng^1, 2, *^

^1^ Key Laboratory of Emergency and Trauma, Ministry of Education, Key Laboratory of Hainan Trauma and Disaster Rescue, Key Laboratory of Haikou Trauma, Department of Wound Repair, The First Affiliated Hospital of Hainan Medical University, Hainan Medical University, Haikou 571199, China.

^2^ Engineering Research Center for Hainan Bio-Smart Materials and Bio-Medical Devices, Key Laboratory of Hainan Functional Materials and Molecular Imaging, Hainan Medical University, Haikou 571199, China.

^3^ Department of Chemistry, Chung-Ang University, Seoul 06974, South Korea.

^+^ These authors contributed equally to this work.

**Corresponding authors**

**E-mail addresses:** [wangrui@muhn.edu.cn](mailto:wangrui@muhn.edu.cn); [jbchoo@cau.ac.kr](mailto:jbchoo@cau.ac.kr); [yufabiao@muhn.edu.cn](mailto:yufabiao@muhn.edu.cn); [chengshaowen1@126.com](mailto:chengshaowen1@126.com)

**Keywords:** Silent-region SERS; Photodynamic-antibiotic combination therapy; Wound infection; Reducing recurrence risk; Real-time monitoring

**Fig. S1** Biocompatibility experiments of SERS Probes. Cytotoxicity assays of the SERS probe in NIH-3T3 (a), HaCaT (b), and HUVEC (c) cells. (d) shows the experimental images of samples at corresponding concentrations, while (e) depicts the histogram of hemolysis test results for the SERS probes at different concentrations.

**Fig. S2** Evaluation of the effect of different treatment conditions on the removal of P. aeruginosa biofilm. Confocal laser scanning microscopy (CLSM) images and corresponding side views of mature P. aeruginosa biofilms after different treatments including (a) PBS, (b) laser irradiation, (c) AuPB NPs with laser irradiation, (d) CS, (e) SERS probe, and (f) SERS probe with laser irradiation. (g) Quantitative analysis of biofilm thickness in a-f. (h) Crystal violet staining images and OD590 quantitative results of P. aeruginosa biofilms formed after treatments. Scale bar: 50 μm (n=3). Statistical analysis was performed using one-way analysis of variance followed by post hoc Tukey multiple comparisons (n = 3, mean ± S. E. M., *P < 0.05, **P < 0.01, ***P < 0.001, ****P < 0.0001).

**Fig. S3** Release behavior of CS from SERS probe and verification of its antibacterial activity after release. (a) Standard curve of the released CS. (b) Cumulative release curve of CS from SERS probe at different time points (0, 3, 6, 12, 24, 48, 72 h). (c) Verification of the antibacterial activity of CS released by SERS probe: the released group and the control group with the same concentration of free CS were applied to E. coli and P. aeruginosa, respectively, and plate culture was performed to obtain bacterial plate photographs. (d) and (e) Quantitative analysis of colony-forming units in (c), respectively.

**Fig. S4** *In vivo* antibacterial efficacy of the SERS probe. The wounds of db/db mice were inoculated with (a) *E. coli* and (b) *P. aeruginosa*, and divided into control, PS, AuPB NPs, CS and SERS probes. The figure showed representative wound images after the different treatments, simulated images of the healing rate (HR), LB plate images, and SERS images of bacterial colonies after the different treatments, respectively. (c, d) Body weight, (e, f) healing rate (e, f), (g, h) plate CFU count, and (I, j) quantitative results of SERS imaging are shown for the db/db mice wound infection model with E*. coli* and *P. aeruginosa*, respectively. Scar bar: 0.5 cm.

**Fig. S5** *In vivo* antibacterial efficacy of the SERS probe in mixed bacterial infection models. The wounds of BALB/c (a) and db/db (b) mice were inoculated with *E. coli* and *P. aeruginosa* and divided into control, PS, AuPB NPs, CS and SERS probes. The images show representative wound images after the different treatments, simulated images of healing rates, LB plate images and SERS images of bacterial colonies after the different treatments, respectively. (c, d) Body weight, (e, f) healing rate, (g, h) plate CFU counts, and (i, j) quantitative results of SERS imaging for (a) BALB/c and (b) db/db mice wound infection models with *E. coli* and *P. aeruginosa*, respectively, are shown. Scar bar: 0.5 cm.

**Fig. S6** Bright-field images of SERS mapping for (a) BALB/c mice models and (b) db/db mice models.

**Fig. S7** Wound healing by enhancing collagen deposition and angiogenesis in bacterial-infected diabetic wounds with SERS probes. db/db mouse models infected with *E. coli*, *P. aeruginosa*, or mixed bacterial infection, followed by different treatments, including PS, AuPB NPs, CS, and SERS probe administration. Histological and immunohistochemical analyses were performed to assess wound healing under different therapeutic strategies at days 2 and 8. (a) HE staining for evaluating tissue structural changes, (b) Masson’s trichrome staining for visualizing collagen fiber deposition, (c) VEGF immunostaining for analyzing angiogenesis, (d) CD31 immunostaining for detecting endothelial cell markers, and (e) α-SMA immunostaining for assessing vascular smooth muscle cell activation. (f-m) Quantitative analysis of staining area ratios corresponding to (a-e). Scale bar = 500 μm.

**Fig. S8** H&E staining was used to evaluate wound healing (magnified). BALB/c mouse models were infected with E. coli, P. aeruginosa, or mixed bacterial infection. Subsequently, different treatments were administered, including PS, AuPB NPs, CS, and SERS probes. Histological analysis was performed on day 2 (a) and day 8 (b). HE staining (scale bar = 500 μm) and corresponding high-magnification images (scale bar = 200 μm).

**Fig. S9** Masson staining was used to evaluate wound healing (magnified). BALB/c mouse models were infected with E. coli, P. aeruginosa, or mixed bacterial infection. Subsequently, different treatments were administered, including PS, AuPB NPs, CS, and SERS probes. Histological analysis was performed on day 2 (a) and day 8 (b). HE staining (scale bar = 500 μm) and corresponding high-magnification images (scale bar = 200 μm).

**Fig. S10** H&E staining was used to evaluate wound healing (magnified). db/db mouse models were infected with E. coli, P. aeruginosa, or mixed bacterial infection. Subsequently, different treatments were administered, including PS, AuPB NPs, CS, and SERS probes. Histological analysis was performed on day 2 (a) and day 8 (b). HE staining (scale bar = 500 μm) and corresponding high-magnification images (scale bar = 200 μm).

**Fig. S11** Masson staining was used to evaluate wound healing (magnified). db/db mouse models were infected with E. coli, P. aeruginosa, or mixed bacterial infection. Subsequently, different treatments were administered, including PS, AuPB NPs, CS, and SERS probes. Histological analysis was performed on day 2 (a) and day 8 (b). HE staining (scale bar = 500 μm) and corresponding high-magnification images (scale bar = 200 μm).

**Table. S1** Clinical wound samples of 36 patients with confirmed bacterial infections

| Patient ID | Gender | Age | Pathogen name | Clinical diagnosis | SERS  Intensity |
| --- | --- | --- | --- | --- | --- |
| P001 | Male | 61 | Staphylococcus aureus (G^+^) | Soft tissue infection | 675 |
| P002 | Male | 75 | Streptococcus agalactiae (G^+^) | Type Ⅱ diabetic foot disease | 252 |
| P003 | Male | 41 | Gemella morbillorum (G^+^) | Local infection of skin and subcutaneous tissue | 764 |
| P004 | Male | 50 | Staphylococcus aureus (G^+^) | Type Ⅱ diabetic ulcer | 724 |
| P005 | Female | 82 | Enterococcus faecalis (G^+^) | Type Ⅱ diabetic foot disease | 666 |
| P006 | Male | 33 | Staphylococcus aureus (G^+^) | Necrotizing fasciitis of the lower leg | 596 |
| P007 | Male | 63 | Streptococcus  pyogenes (Group A) (G^+^) | Type Ⅱ diabetic foot disease | 739 |
| P008 | Male | 35 | Streptococcus agalactiae (G^+^) | Type Ⅱ diabetic ulcer | 428 |
| P009 | Male | 68 | Corynebacteriun striatum (G^+^) | Type Ⅱ diabetic foot disease | 736 |
| P010 | Male | 61 | Multidrug-resistant Corynebacterium striatum (G^+^) | Type Ⅱ diabetic foot disease | 223 |
| P011 | Male | 54 | Enterococcus faecalis (G^+^),  Staphylococcus aureus (G^+^) | Type Ⅱ diabetic foot disease | 567 |
| P012 | Female | 92 | Enterococcus faecalis (G^+^) | Post-traumatic wound infection | 742 |
| P013 | Male | 66 | Multidrug-resistant Staphylococcus aureus (G^+^) | Lower Limb Soft Tissue Infection | 695 |
| P014 | Male | 62 | Staphylococcus aureus (G^+^) | Type Ⅱ diabetic foot disease | 809 |
| P015 | Male | 44 | Streptococcus agalactiae (G^+^) | Type Ⅱ Diabetic Necrotizing Fasciitis | 629 |
| P016 | Female | 71 | Streptococcus dysgalactiae (G^+^) | Type Ⅱ diabetic foot disease | 415 |
| P017 | Male | 41 | Actinomyces europaeus (G^+^) | Lower Limb Soft Tissue Infection | 674 |
| P018 | Male | 95 | Multidrug-resistant Enterococcus faecium (Group D) (G^+^) | Stage IV Sacrococcygeal Pressure Ulcer | 718 |
| P019 | Male | 74 | Staphylococcus aureus (G^+^) | Skin Infections | 479 |
| P020 | Male | 50 | Multidrug-resistant Staphylococcus aureus (G^+^) | Type Ⅱ diabetic foot disease | 353 |
| P021 | Female | 75 | Enterococcus faecalis (G^+^) | Type Ⅱ diabetic foot disease | 838 |
| P022 | Female | 56 | Multidrug-resistant Acinetobacter baumannii (G^-^) | Type Ⅱ diabetic foot disease | 3541 |
| P023 | Male | 71 | Pseudomonas aeruginosa (G^-^), Proteus hauseri (G^-^) | Type Ⅱ diabetic foot disease | 1927 |
| P024 | Male | 60 | Enterobacter cloacae complex (G^-^) | Type Ⅱ diabetic foot disease | 1413 |
| P025 | Male | 69 | Proteus vulgaris (G^-^) | Type Ⅱ diabetic foot disease | 1501 |
| P026 | Male | 67 | Multidrug-resistant Acinetobacter baumannii (G^-^) | Type Ⅱ diabetic ulcer | 1614 |
| P027 | Female | 66 | Multidrug-resistant Klebsiella pneumoniae (G^-^) | Localized Skin and Subcutaneous Tissue Infections | 1417 |
| P028 | Male | 65 | Multidrug-resistant Proteus mirabilis (G^-^) | Type Ⅱ diabetic foot disease | 1068 |
| P029 | Male | 79 | Pseudomonas aeruginosa (G^-^), Multidrug-resistant  Escherichia coli (G^-^) | Stage IV Pressure Injury in Weight-Bearing Areas | 1149 |
| P030 | Female | 89 | Klebsiella pneumoniae (G^-^) | Stage IV Sacrococcygeal Pressure Ulcer | 1184 |
| P031 | Female | 59 | Pseudomonas aeruginosa (G^-^), Multidrug-resistant Enterobacter cloacae complex (G^-^) | Type Ⅱ diabetic foot disease | 2009 |
| P032 | Female | 84 | Proteus mirabilis (G^-^) | Type Ⅱ diabetic foot disease | 1591 |
| P033 | Female | 54 | Pseudomonas aeruginosa (G^-^) | Localized Skin and Subcutaneous Tissue Infections | 1905 |
| P034 | Female | 47 | Aeromonas hydrophila (G^-^), Pseudomonas aeruginosa (G^-^), Multidrug-resistant Enterococcus faecium (Group D) (G^+^) | Systemic Lupus Erythematosus | 2297 |
| P035 | Male | 62 | Enterococcus faecium (G^+^), Morganella morganii  ssp morganii (G^-^) | Type Ⅱ diabetic foot disease | 1577 |
| P036 | Male | 67 | Corynebacterium tuberculostearicum (G^-^) | Necrotizing Fasciitis | 1235 |
